# Structural Characterization of Biomaterials by Means of Small Angle X-rays and Neutron Scattering (SAXS and SANS), and Light Scattering Experiments

**DOI:** 10.3390/molecules25235624

**Published:** 2020-11-29

**Authors:** Domenico Lombardo, Pietro Calandra, Mikhail A. Kiselev

**Affiliations:** 1 CNR-IPCF, Consiglio Nazionale delle Ricerche, Istituto per i Processi Chimico-Fisici, 98158 Messina, Italy; 2 CNR-ISMN, Consiglio Nazionale delle Ricerche, Istituto Studio Materiali Nanostrutturati, 00015 Roma, Italy; pietro.calandra@ismn.cnr.it; 3 Frank Laboratory of Neutron Physics, Joint Institute for Nuclear Research, Dubna, 141980 Moscow, Russia; kiselev@jinr.ru

**Keywords:** small angle X-rays scattering (SAXS), small angle neutron scattering (SANS), light scattering, inter-particle interactions, nanotechnology, biomaterials characterization

## Abstract

Scattering techniques represent non-invasive experimental approaches and powerful tools for the investigation of structure and conformation of biomaterial systems in a wide range of distances, ranging from the nanometric to micrometric scale. More specifically, small-angle X-rays and neutron scattering and light scattering techniques represent well-established experimental techniques for the investigation of the structural properties of biomaterials and, through the use of suitable models, they allow to study and mimic various biological systems under physiologically relevant conditions. They provide the ensemble averaged (and then statistically relevant) information under in situ and *operando* conditions, and represent useful tools complementary to the various traditional imaging techniques that, on the contrary, reveal more local structural information. Together with the classical structure characterization approaches, we introduce the basic concepts that make it possible to examine inter-particles interactions, and to study the growth processes and conformational changes in nanostructures, which have become increasingly relevant for an accurate understanding and prediction of various mechanisms in the fields of biotechnology and nanotechnology. The upgrade of the various scattering techniques, such as the contrast variation or time resolved experiments, offers unique opportunities to study the nano- and mesoscopic structure and their evolution with time in a way not accessible by other techniques. For this reason, highly performant instruments are installed at most of the facility research centers worldwide. These new insights allow to largely ameliorate the control of (chemico-physical and biologic) processes of complex (bio-)materials at the molecular length scales, and open a full potential for the development and engineering of a variety of nano-scale biomaterials for advanced applications.

## 1. Introduction

With recent advances in experimental and theoretical approaches in material science and biotechnology, a deeper understanding of the structure-function relationships and their implication in advanced applications has been made possible [1,2,3,4,5]. Novel nanostructured systems prepared through chemical synthesis or self-assembly approaches find applications in various biotechnology fields include drug/gene delivery [6,7,8,9,10,11], tissue engineering [12,13,14,15] and nanomedicine [16,17,18,19]. More specifically, a relevant number of both natural products and new developed nanostructures are hierarchically organized and composed of structure-building elements (so called “building blocks”), which consist of nano-sized (bio-)molecules self-assembled into supramolecular aggregates, or at materials systems interfaces [20,21,22,23,24]. The characterization of the structural features and the elucidation of the self-assembly processes occurring in those materials is important information requested when studying the relationships between physical properties and specific desired functions. In this respect, scattering techniques represent powerful methods for obtaining useful information on size, shape and morphological transitions occurring in nano-structured materials. A detailed description concerning how basic components of new materials are structured and interact with each other enables the convenient manipulation of their relevant properties for the design and the engineering of novel bio-nanotechnologies [25,26,27,28,29,30]. In this review, we introduce the basic concepts of scattering methods and their data interpretation, as well as of the complementarity of the information produced by the different types of radiation (e.g., neutrons, X-ray or light), while presenting different examples of recent applications and developments in the field of bio-nanotechnology.

## 2. Basic Principles of the Scattering Techniques

The scattering technique has been proven to be a unique and powerful tool for elucidating the structure, inter-particles interaction and phase transitions in nanostructured materials and complex systems [31,32,33].

X-ray photons interact with all the electrons in the material under investigation, while the partial destructive interferences among the waves scattered by the particular distribution of electrons (orbitals) around the nucleus strongly influence the atomic scattering factor f, which depends on the X-ray energy and decreases with the increasing scattering angle θ.

Neutron scattering by matter arises through (short-range) nuclear interactions, or magnetically, as neutron magnetic moment couples with the magnetic induction B, while the scattering length *b_i_* depends on the nature of the nuclei of the specific atoms (with no relation to the atomic number). Neutrons also interact with atoms that have unpaired electron spins, via a magnetic dipole interaction.

Optical photons, which have energies much lower than X-rays, are scattered only by the outer part of the electronic cloud of an atom, while the scattering length density is proportional to the polarizability of the materials. More specifically the deformation of the electron cloud, due to the electric field component of the electromagnetic (laser) radiation, produces and induced dipole momentum *P*(*t*) = *αE*(*t*) (where *α* is the polarizability). The resulting charge movement radiates (scatters) light.

**Figure 1:**
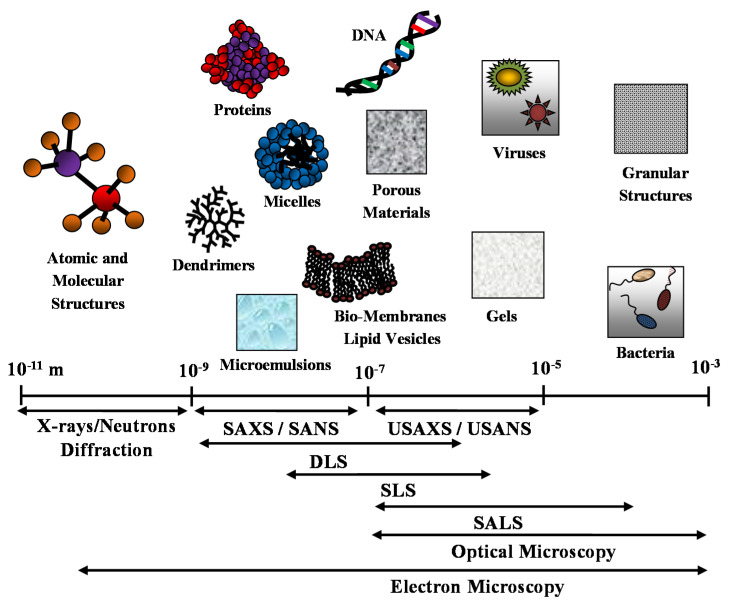
Different space resolution of the main scattering techniques: small angle X-rays and neutrons scattering (SAXS and SANS), ultra-SAXS and ultra-SANS (USAXS and USANS) and dynamic, static and small angle light scattering (DLS, SLS and SALS). Comparison with complementary optical and electron microscopy techniques.

It is worth noting that optical and electron microscopy techniques (such as transmission electron microscopy (TEM) and scanning electron microscopy (SEM)), together with diffraction techniques, are useful complementary methods that can enrich and support the structural description of the nanostructured (bio-)material system under investigation. In Figure 1, we report the different space resolution of the scattering techniques and compare it with complementary microscopy and diffraction techniques.

However, while microscopic techniques acquire the structural information in real (direct) space, scattering data are recorded in reciprocal (Fourier) space. For this reason, the structural information obtained by scattering experiments should be obtained starting from suitable modeling approaches of the scattering data in the reciprocal space, or from the density distribution profile (i.e., *P*(*r*) versus *r* plot) in real space, obtained by a Fourier transform of the scattering length density [31,32]. Moreover, contrary to electron microscopic techniques, where a preliminary sample treatment (such as drying, freezing, sectioning or staining) is often required, scattering techniques are non-destructive methods capable to furnish statistically representative structural information of a sample in a wide range of conditions of the investigated samples.

## 3. Small Angle Scattering of Neutrons (SANS) and X-rays (SAXS)

**Figure 2:**
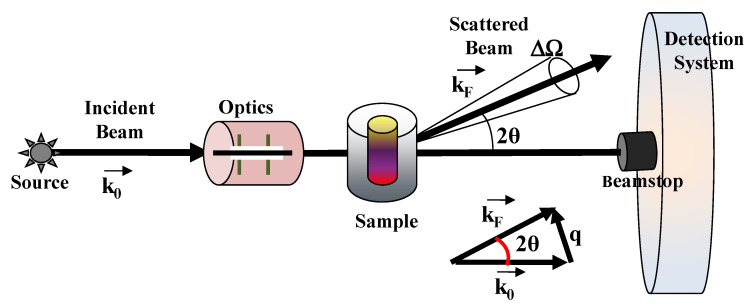
Typical scattering geometry for small angle scattering (SAS) experiments. An incident radiation beam from a neutrons or X-rays source impinging on the material system (sample) under investigation is scattered at a given scattering angle 2θ and is collected by a proper detection system.

Small-angle scattering of neutron (SANS) and X-rays (SAXS) probe the statistical ensemble of the nano-structures and deal with the diffusion of electromagnetic or particle waves (with a given wavelength λ) by heterogeneities in matter [31,32,33,34]. An example of a typical scattering geometry is reported in Figure 2. In a small-angle scattering (SAS) experiment, a collimated, monochromatic beam of the incident radiation impinges on a scattering cell (illuminated volume) containing the sample under investigation. The scattering intensity at various scattering angles θ is collected by a detector, placed at a given distance from the sample. The detector is shielded from the primary beam by an active beamstop, which records the transmitted beam intensity for later use in normalization procedures. The difference between the scattered (*k_F_*) and incident (*k*_0_) wavevectors furnishes the scattering wavevector *q* = |*k_F_* − *k*_0_| = (4π/λ)sin(θ), which has the dimensions of a reciprocal length (common units are Å^−1^ or nm^−1^).

### 3.1. SAXS and SANS Experimental Approaches and Technical Features

#### 3.1.1. Experimental Setup and Preliminary Data Treatment

In the SAXS laboratory setup, the X-rays source is given by a conventional X-ray tube, which deliver the wavelengths (λ) of the most intensive *K_α_* lines of the target anode. Commonly used materials for the anode are copper (λ = 1.54 Å) and molybdenum (λ = 0.71 Å) [31,32].

Alternatively, a synchrotron SAXS beamline can be developed starting from the high photon flux emitted by a wiggler, or an undulator of a syncrotron radiation facility. By using a monochromator, a single wavelength can be selected from the emitted polychromatic synchrotron radiation spectrum. Concerning the SAXS technique (which typically operates at scattering angle between 0.1°–5°), it is possible to set a further detection system on the same experimental set-up, which allows wide angle X-ray scattering (WAXS) experiments (at a scattering angle of typically >5°). The WAXS technique cover a wide range of scattering variables down to small d-spacing, which corresponds roughly to the size of chemical bonds, and provides useful complementary information of sub-nanometer-sized structures [31,32]. The WAXS technique is largely employed for the investigation of the degree of crystallinity of polymer samples. It can also be used to determine the phase (or chemical) composition of the texture of a film (with the determination of sizes and preferred alignment of crystallites), as well as the structural organization of hydrocarbon chain packing and lamellar phases (and their crystalline-like ordered phases, when present) in lipid-based bio-membrane systems [31,32].

Concerning the SANS experimental setup, two main sources of neutrons are employed, namely the steady-state reactors (where neutrons are continuously produced by fission processes) and the spallation sources (where a pulsed neutron beam, typically with a 25 Hz or 50 Hz frequency, is generated by the collision of high-energy protons which chop off heavy atoms) [33,34].

In a typical SAS experiment, part of the incident beam is absorbed in the material system. For this reason, the number of photons scattered in the solid angle ΔΩ in the direction 2θ have to be normalized, with respect to the number of ph otons transmitted through the sample. The photons that pass through the sample without being s cattered are captured by an active beamstop, and monitored to determine the specimen transmission *T*, while the scattered intensity is normalized by the transmission. The scattering experiment intensity is connected with the differential scattering cross section *d*Σ/dΩ(*q*) per unit volume *V* (illuminated sample volume). This quantity, which is obtained from count rates coming from specific detection systems, represent the scaled ‘absolute intensity’ (measured in cm^−1^), and is expressed as a function of the flux (number of photons per second in the solid angle ΔΩ) incoming on the sample *C*_0_ and collected at the detector *C*.

(1) $$I_{Abs}\;=\;\frac{1}{V}\frac{d\sigma \left(q\right)}{d\Omega }\;=\;\frac{C}{C_{0}\Delta {\Omega Te}_{S}}$$

where *T* is the sample transmission and *e_S_* is the sample thickness.

#### 3.1.2. Atomic Scattering Length and Scattering Length Density

The main difference between the SAXS and SANS methods originates from the fact that neutrons directly interact with atomic nuclei, while X-ray photons scatter from electrons of the reference material system. This feature is reflected by the scattering length: for neutrons, b assumes specific values for each element (with no relation to the atomic number). In contrast, in the case of X-ray scattering, b is proportional to the number of electrons in an element (except for wavelengths close to an atom absorption edge).

In the case of the neutron-nucleus scattering, the strength is described by the total neutron scattering cross-section, which is a measure the effective cross-sectional area of the neutron-nucleus interaction potential, and is contributed from both coherent and incoherent scattering. Coherent scattering comprises diffraction and small angle scattering (SAS) and has a counterpart in X-ray scattering. Incoherent scattering arises from a unique interaction of the neutron with the momentum of the nucleus and has no equivalent with either X-ray or light scattering. The neutron scattering lengths *b_i_* for individual atoms, that depends on the specific neutron-nucleus interaction strengths, are not easily predictable theoretically, and specific experiments are required to tabulate those values [31,33,35]. For X-rays, as the scattering power scales with electron density in the material, one can obtain tabulated values of the atomic scattering factors [31,35].

Depending on the type of radiation, the scattering from assemblies of atoms and molecules is most conveniently described in terms of their scattering length density (SLD), which describes how (on average) the component molecules that effectively scatters the incident radiation. The averaging SLD calculation consists in the summation of the atomic scattering length *b_i_* of each atom over a volume that is representative of a macromolecule (or a phase region), where the scattering features of the material do not vary. More specifically, the SLD from a (macro-)molecule containing *x_i_* atoms i is given by *ρ_i_ = ∑x_i_b_i_/v_m_*, (where the sum is extended to the scattering length contributions *b_i_* from the different *N* atoms contained in the molecular volume *V_m_*). From the knowledge of the material bulk density *ρ_B_* and the molecular weight M, the molecular volume can be computed as *V_m_* = *M*/*ρ_B_N_a_* (where *N_a_* is the Avogadro constant).

Detailed information of the neutron scattering length can be found in the literature [35]. In Table 1, we report the SANS and SAXS coherent scattering lengths *b_i_* of some relevant elements of biological systems, while, in Table 2, we report the SANS and SAXS scattering length density (SLD) for H_2_O and D_2_O molecules [31].

#### 3.1.3. The Contrast Variation Method

**Figure 3:**
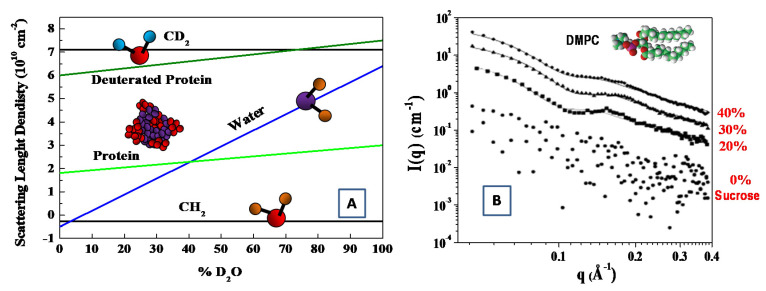
Scattering length density versus percentage of D_2_O in solution for protein and CH_2_ molecules (**A**) (adapted from Jacrot [38]). SAXS curves at *T* = 30 °C for extruded dimyristoylphosphatidylcholine (DMPC) vesicles in the aqueous solutions with sucrose concentrations of 0% (circles), 20% (squares), 30% (triangles) and 45% (rhombuses) (**B**). (adapted from Kiselev et al. [45]).

As deuterium has a much larger coherent neutron scattering length than hydrogen (see Table 1). Deuterium (^2^H)-isotope labeling and contrast variation have been employed in SANS for probing the structural information of biological molecules [33,36]. More specifically, by changing relative ratio of D_2_O/H_2_O, the scattering length density can vary, to a wide extent, to match the scattering length density of different materials. In this case, the structural information of the individual components (within a multi-component biological complexes) can be best probed through selective deuterium labeling, when the scattering of the other unlabeled components is rather weak. Such an approach has been employed to investigate complex interaction in bio-systems, such as multi-protein complexes, protein–carbohydrate complexes, protein–lipid complexes [36,37]. The structural information of the individual components of biomolecules can be probed by means of the contrast variation (or scattering length density matching) through the variation of the D_2_O ratio in solution (see Figure 3) [38].

The contrast variation is an approach that can be applied in a profitable way also in X-ray scattering experiments. In contrast to traditional X-ray sources, which work at a limited set of fixed wavelengths, the use of synchrotron radiation allows one to select a single wavelength (or energy) by means of a monochromator. For wavelengths close to the absorption edge, for which the atom strongly absorbs radiation, the X-ray scattering factor f (which is roughly proportional to the number of electrons of the reference atom) undergoes a variation, due to anomalous dispersion. The energy dependent scattering factor can, therefore, be best described as the complex number f(E) = Z + f′(E) + if″(E) (where Z is the atomic number, f′(E) is the anomalous scattering factor, and f″(E) is a factor accounting for absorption effects). This approach, called anomalous small-angle X-ray scattering (ASAXS) [39], makes it possible to vary, and then ‘highlight’, the scattering factor (up to about 20%) for one particular element in the sample under investigation. Combined SAXS and SANS contrast variation experiments have been employed to resolve the details of the complex morphology of Sulfonated PEEK membranes for fuel cells applications [40]. The ASAXS method was employed to localize the dibromophenol (DPB) guest molecules in a system constituted of DPPC/water multilamellar liposomes [41]. In the case of the higher DBP concentration, the distribution of the guest molecules, which was characterized by a sharp function, highlighted the special role played by the DBP molecules in the organization of the interdigitated multilamellar phase. ASAXS method has also been employed for the microstructure characterization of charged soft nanomaterials, by using high-brilliance synchrotron radiation [39,42]. Examples include flexible/stiff chain and brush-like) polyelectrolytes, colloids, DNA, RNA and polysaccharides, where the spatial distribution of free and bound counterions around a macroion could be determined with high precision (and compared with theoretical models) by tuning the energy in the vicinity of the absorption edge of the counterions [39,42].

Finally, with the aim to increase the X-ray’s contrast, several electron-rich small molecules have been used to modify the solvent electron density, including sucrose [43,44,45,46,47], glycerol [48,49] and salt [50]. For instance, the increase in the X-ray’s contrast has been obtained with the suitable addition of sucrose during the investigation of a water solution of dimyristoylphosphatidylcholine (DMPC) phospholipid vesicles [45]. In that case, the region of sucrose concentrations between 30% and 40% created the best experimental conditions for SAXS experiments, while no perturbing influence of sucrose on the membrane thickness or mutual packing of hydrocarbon chains was detected [45,46]. This powerful approach has also been used to study the structure and flexibility of an anionic dual-surfactant wormlike micelles in solution [47].

In summary, the variation of the scattering cross section of elements across the periodic table is very different for X-rays and neutrons. The X-rays scattering cross section is proportional to the electron density, and increases with the atomic number, while the variation of the X-ray scattering factor f (due to anomalous dispersion) makes it possible to highlight, and then to “identify”, specific elements in the sample under investigation. On the other side, the neutrons scattering cross section varies irregularly with the atomic number, while isotopes of the same element may have completely different neutron scattering cross-sections. This isotopic sensitivity may be used in contrast variation measurements, which allow neutrons to distinguish the different isotopes in a macromolecular structure [51,52]. However, whereas, in the ASAXS method, only a single sample is needed and experiments are carried out near the X-ray absorption edge of the element of interest, in a contrast variation SANS experiment, several samples with different contents of isotopes (such as deuterium) should be prepared, and the technique is a bit more laborious.

#### 3.1.4. Complementary Aspects of SAXS and SANS Techniques

It is worth pointing that even the rotary anode X-ray source in regular laboratories possesses an X-ray flux of 10^11^ photons/cm^2^/s, which is several orders of magnitude higher than flux of neutrons (10^8^–10^9^ neutrons/cm^2^/s) produced by nuclear fission in reactor-based neutron sources (continuous source), or by spallation in accelerator-based neutron sources (pulsed sources). For this reason, SANS requires a higher concentration and larger (hundreds of μL) volumes of the investigated material sample, as well as a longer measurement time to acquire statistically significant scattering signals, compared to the SAXS techniques (which require tens of μL volumes). Due to the high flux of the X-ray beam from synchrotron radiation facility (and the combination with modern 2-D fast detectors), it is possible to perform fast detection of the SAXS spectra down to the millisecond resolution. With a flux density, even higher than 1 × 10^17^ photons/sec/mm^2^, synchrotron radiation SAXS can be used for time-resolved experiments, for the investigation of fast (structural/dynamic) transitions (up to the sub-millisecond resolution) encountered in biotechnology and material science processes. Kinetic studies are much less feasible with SANS, especially if the sample contains low scattering objects, which is a frequent feature of most biomaterials. Then, for time resolved SANS experiments, high-flux neutrons (spallation sources or high-flux reactors), good scatterers, slow reaction, and narrow Q-range are required. However, SANS is much less harmful and non-destructive for most biomaterials, while X-rays exhibit better penetration (that increases with photon energy), which can lead to sample damage or degradation (such as radiolysis, solvated electron, etc.) or induce chemical changes (such as free radical formation), or can alter the state of a sample over the acquisition time (such as the aggregation due to cross-linking or temperature increase). SANS also allows for detecting the scattering at lower angles as compared to SAXS, thus, providing the observation of larger scattering particles (usually >1000 Å for SANS and ∼500–600 Å for SAXS). Finally, sample preparation in H_2_O represent another practical advantage of (synchrotron) SAXS techniques, while, due to contrast, SANS samples must be prepared in D_2_O. However, SANS opens wider possibilities to elucidate different structural features of multicomponent hydrogels by using the contrast variation technique (by varying ratio of D_2_O/H_2_O in the sample) [33,34,35,36].

### 3.2. Form Factor P(Q) Analysis

At small scattering angles 2θ, the (coherent) scattering cross-section per particle in the so called “static approximation” can be expressed is terms of the scattering length *b_i_* of the particle that occupies the position R in the material system [38,53].

(2) $$\frac{d\sigma }{d\Omega }=\frac{1}{N}{\left[\underset{j}{\sum }b_{j}e^{iqR_{j}}\right]}^{2}$$

For a small momentum transfer (continuum approximation), we can express scattering functions in terms of the scattering length density (SLD) *ρ*(*r*) = Σ *ρ*(*r*)*_i_b_i_*, where *ρ*(*r*)*_i_* is the local density of scatterers of type *i* [38,53]. In the SANS experiments, the scattering length density is written as *ρ*(*r*) = Σ*_i_*(*b_i_n_i_*(*r*)), where *b_i_* is the scattering length of nucleus of type *i*, and *n_i_*(*r*) is the corresponding number density of such nuclei. For SAXS experiments, *ρ*(*r*) = (*e*^2^/*mc*^2^)*n_el_*(*r*), where (*e*^2^/*mc*^2^) is the electron Thompson scattering length and *n_el_*(*r*) is the electron number density.

By replacing *b_i_* by a locally averaged scattering length density *ρ_i_*(*r*) (where r is a variable position vector), we can integrate over the irradiated sample volume *V*, and obtain the basic expression to model scattering functions of specific systems.

(3) $$\frac{d\sigma }{d\Omega }=\frac{1}{N}{\left[\int_{V}\left(\rho_{i}\left(r\right)exp\left(iqr\right)d^{3}r\right)\right]}^{2}$$

For a system composed of nearly monodisperse particles in solution, the SAXS scattering intensity *I*(*q*) = (*dσ/d*Ω) can be expressed as a product of the form factor *P*(*q*), which contains information on the size, morphology and molecular mass of the scattering particles, and the structure factor *S*(*q*), describing the inter-particle interaction [31,32].

(4) $$I\left(q\right)=N{\left(\Delta \rho \right)}^{2}P\left(q\right)S\left(q\right)$$

where *N* is the number density of the particles, and Δ*ρ* = (*ρ* − *ρ*_0_) is the so-called “contrast” (i.e., the difference between the scattering length density of the particle *ρ* and that of the solvent *ρ*_0_). In the diluted region, the interaction between the nano-particles composing the sample can be neglected (i.e., *S*(*q*) ~1), so that the analysis of scattering intensity *I*(*q*) can furnish direct information of the morphological features of scattering particles through the analysis of the scattering form factor *P*(*q*).

General expressions of the form factor *P*(*q*) are known for a wide range of different shapes, such as homogeneous spheres, core-shells spheres, cylinders, concentric cylinders, discs, ellipsoids (or ellipsoidal shells) [32,53,54]. For a uniform sphere (radius *R*_0_ and volume *V*) with excess electron density Δ*ρ*, the corresponding equation for the form factor is given by [31,32].

(5) $$P\left(q\right)={\left(\Delta \rho \right)}^{2}V_{0}^{2}{\left[3\frac{\sin\left(qR_{0}\right)-qR_{0c}\cos\left(qR_{0}\right)}{{\left(qR_{0}\right)}^{2}}\right]}^{2}$$

**Figure 4:**
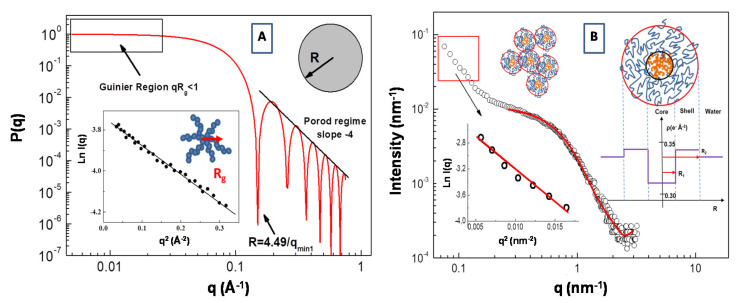
Plot of the form factor *P*(*q*) of a sphere with *R* = 30 Å. An example of a SAXS Guinier fitting of *P*(*q*) of a star polymers nanopartarticle (with a radius of gyration *Rg* = 17.2 Å) in water solution is presented in the inset (**A**). Fit of the form factor *P*(*q*) SAXS intensity for the polydimethylsiloxane-b-polyethyleneoxide (PDMS-b-PEO)/water. System (at ca oncentration of 0.05 g/cm^3^) with the core-shell model (**B**). Scattering-length density (SLD) distribution of the core-shell micelles is reported in the inset (adapted from Lombardo et al. [61]).

An example of the form factor of a uniform sphere with radius *R* = 30 Å is reported in Figure 4A. Expanding the form factor *P*(*q*) in the low q range (i.e., in the so called Guinier region for *qR_g_* < 1), the particle form factor can be expressed as *P*(*q*) = *P*(0)*exp*(−*q*^2^*R_g_*^2^/3). In this case, information about the particles radius of gyration *R_g_* can be obtained from the slope of the representation *lnI*(*q*) vs. *q*^2^ (in the low q region of the SAS spectra, i.e., *qR_g_* < 1). This procedure is depicted in the inset of Figure 4A, where an SAXS Guinier fitting of *P*(*q*) of a star branched polymer nanopartarticle in water solution (with *R_g_* = 17.2 Å) is presented.

A large variety of nanostructured materials employed in biomedical and nanomedicine applications, are based on the employment of amphiphilic polymers (and block copolymers), which are able to encapsulate therapeutic drugs via self-assembly processes [55]. Amphiphilic block copolymers in a selective solvent (like water) form micellar aggregates composed of a core of collapsed hydrophobic segments (which can incorporate hydrophobic drugs) and an outer shell of hydrophilic copolymer brushes (which provides steric stability and flexibility for further functionalities). This core-shell architecture is strongly influenced by thermodynamic parameters (such as polymer lengths and ratios of the polymers blocks), as well as the kinetic control of the self-assembly conditions [55,56,57,58], and has been considered as one of the desired structures for sustained release and targeted drug delivery [59,60]. In this respect, the knowledge of the micelle structures, as determined by SAS experiments, will provide useful insights for the design and engineering of polymeric drug delivery systems.

In Figure 4B, we report the SAXS profile for the aqueous solution of amphiphilic polydimethylsiloxane-b-polyethyleneoxide (PDMS-b-PEO) diblock copolymer. Assuming that block copolymer micellar aggregates are composed of a PDMS core and the PEO chains in a surrounding shell, the corresponding form factor *P*(*q*) can be described by a core-shell model expressed as a function of core and shell radius, *R*_1_ and *R*_2_, respectively, and the core, shell and water scattering-length, *ρ*_c_, *ρ*_s_, *ρ*_0_, respectively [61].

(6) $$P\left(q\right)=\frac{k_{0}}{V_{S}}{\left\{3V_{C}\left(\rho_{C}-\rho_{S}\right)\frac{\left[\sin\left(qR_{1}\right)-qR_{1}\cos\left(qR_{1}\right)\right]}{{\left(qR_{1}\right)}^{3}}+3V_{S}\left(\rho_{S}-\rho_{0}\right)\frac{\left[\sin\left(qR_{2}\right)-qR_{2}\cos\left(qR_{2}\right)\right]}{{\left(qR_{2}\right)}^{3}}\right\}}^{2}+bkg$$

where *k*_0_ is a scale factor and *bkg* is the instrumental background. The scattering-length density is expressed as *ρ =* Σ*_i_b_i_/V*, where *V* is the volume of the core or shell region, while *b_i_* is the scattering length of the corresponding component atoms. In our case, we have a scattering-length density of *ρ*_PEO_ = 0.350 (for PEO), *ρ*_PDMS_ = 0.316 (for PDMS) blocks (monomers) and of *ρ*_H2O_ = 0.334 for the water. Analysis of the SAXS profile according the core-shell model (in the large q region) furnish a value of radius of *R*_1_ = 1.80 nm for the core and *R*_2_ = 4.0 nm for the shell. These micellar aggregates coexist with larger aggregates with an average gyration radius of *R_g_* = 12.3 nm (evidenced by the Guinier analysis in the low *q* region of the SAXS spectrum), which are probably generated by the aggregation process between small micelles (inset of Figure 4B). It is worth noting that the high hydration of the hydrophilic PEO chains produces the shift of the corresponding effective SLD toward that of the solution medium (water). This causes an overall decrease of scattering contrast that causes a correlated attenuation scattering intensity, which can be compensated by the employment of a high intensity synchrotron radiation source for X-rays [62,63]. It is worth pointing out that, in many copolymer micellar systems, there is not a sharp transition (in the values of the effective SLD) at the interface between the shell region and the solvent (water) phase, due to strong hydration regime at the interfaces (as in the case of the high hydration of the PEO chains). In this case, a more realistic SLD distribution can be described by a progressive decay of the density profile of the shell as function of the radial distance r. For example, the so-called “cap and gown” model [64], assumes that the polymer volume fraction is distributed as a (uniform) constant core (cap) and a shell with a decaying density profile (gown), given by the function *φ_P_*_(*r*)_ = *exp*(−*r*^2^/*σ*^2^), described as a function of a the Gaussian width σ. Moreover, Pedersen and Gerstenberg introduced a model for the form factor of block copolymer micelles, consisting in a homogeneous spherical micelle core and Gaussian polymer chains attached to the surface [65].

Another important aspect in the modelling of the form factor *P*(*q*) scattering curves concerns the particle’s polydispersity. As most colloidal and nanoparticles systems are composed of polydisperse nanostructures in solution, the exact form factor of monodisperse particles can be smeared with specific size distribution function *f*(*R_A_*, *r*, *σ*), around an average size value *R_A_* (where *σ* is a parameter characterizing the width of the distribution function). The Schultz distribution, Gaussian or log-normal functions have been often used to represent the size spread (polydispersity) around an average value *R_A_*. For example, the effects of the rectangular and Schultz distributions on the inter-particle structure factor *S*(*q*) has been observed by Kotlarchyk and Chen [66], in SANS experiments on polydisperse interacting colloids.

SANS and SAXS structure factor analyses represent some of the most important experimental approaches for the structural investigation of bio-membranes and mixed lipids complex systems [34,54]. More specifically, the self-assembly formation processes of different nanostructures with different topologies can be efficiently evidenced by the analysis of the SAXS and SANS form factor, thus, highlighting the important role of relevant molecular conformations in many different processes of life science. Particularly interesting is the study of (thermodynamic equilibrium) phases in biological membranes that request specific scattering density profile (SDP) modeling approaches in order to rationalize the rich morphology of lipid assemblies in solution [34,54]. In a study Kiselev et al. [67], a Gaussian distribution has been used to interpret the SANS macroscopic cross-section for a aqueous solution of a 1,2-dipalmitoyl-sn-glycero-3-phosphocholine (DPPC) lipid system in presence of sodium deoxycholate (NaDC) bile salt. In that case, in order to take into proper account the polydispersity, the form factor model of concentric ellipsoids of revolution has been smeared by introducing a Gaussian distribution of the vesicles radii (with a standard deviation σ and average vesicle radius <R>) [67].

### 3.3. Indirect Fourier Transform Method: Pair Distance Distribution Function (PDDF)

An alternative to the fitting approach of the SAS data by analytical functions is obtained by means of an inverse Fourier transformation (IFT) of the scattering curves [68,69,70]. This approach furnishes a pair distance distribution function (PDDF) *p*(*r*), which is represented by using a set of basic kernel functions (such as the splines functions), and gives useful information about the shape-size relationship of the scattering nano-objects (in real space) [68,69,70]. For example, globular particles (such as proteins) yield bell-shaped profiles with a maximum at approximately *D*_max_/2 (where *D*_max_ is the extension of the distribution). On the other hand, in the case of intra- and inter-subunits connected at a given distances, the *p*(*r*) profile presents multiple shoulders and oscillations. As there is no (a priori) hypothesis on the size, shape and polydispersity of the scattered objects, this approach frequently needs to be compared with calculated SAS form factor models (such as spheres, cylinders, vesicles, etc.) and eventually with the complementary information obtained by using other structural characterization techniques (such as microscopy or dynamic light scattering experiments). Generally, the calculation of the *p*(*r*) is not straightforward, due to the limited range of scattering wavevectors (from *q*_min_ to *q*_max_), while the detailed structural information can be obtained with the use of suitable models based on an analytical form for the objects (having complex shapes and inhomogeneous density).

The excess scattering density (form factor) can then be obtained from *p*(*r*) by the so-called square-root deconvolution method [68], while ab initio analysis of particle shape and domain structure programs has been developed by D. Svergun on the basis of IFT approaches [69]. Although this method is used to interpret the form factor *P*(*q*), in the absence of inter-particles interaction (i.e., when the structure factor *S*(*q*) ≈ 1), a recent approach based on the generalized indirect Fourier transform (GIFT) makes it possible to determine simultaneously the structure factor and the form factor (in the presence of inter-particles interaction) [70].

**Figure 5:**
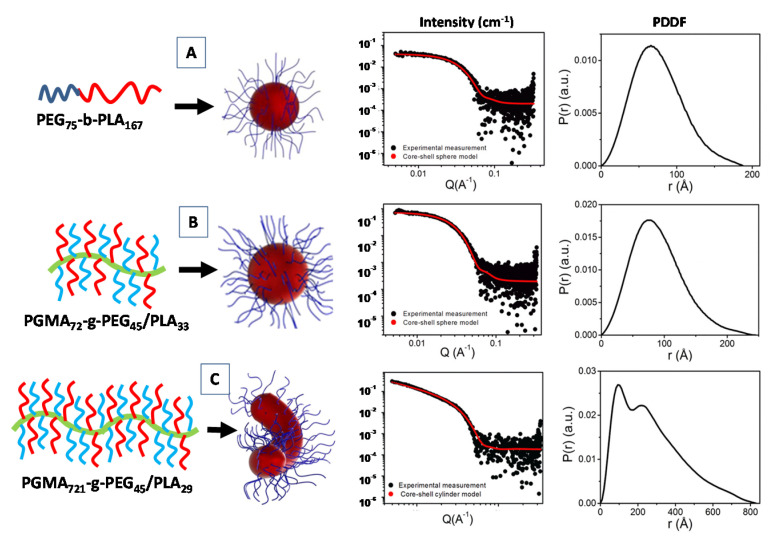
Structural characterization of the SAXS form factor *P*(*q*) and pair distance distribution function (PDDF) analysis for the poly(ethylene glycol)-b-poly(lactic acid) (PEG_75_-b-PLA_167_) diblock copolymers system (**A**), and the poly(ethylene glycol)-b-poly(lactic acid)-b-poly(ethylene glycol) PGMA_72_-g-PEG_45_/PLA_33_ (**B**) and PGMA_721_-g-PEG_45_/PLA_29_ (**C**) graft copolymer systems. Adapted with permission from [71], Copyright 2019 American Chemical Society.

Recently, a SAXS investigation of the micellar nanostructures composed of linear diblock copolymers poly(ethylene glycol)-b-poly(lactic acid) (PEG-b-PLA), as well as a heterografted brush consisting of a poly(glycidyl methacrylate) backbone with PEG and PLA branches (PGMA-g-PEG/PLA) were proposed, by combining the core-shell form factor analysis IFT method [71]. For the simple linear diblock copolymer PEG_75_-b-PLA_167_ the fitting the scattering data (form factor of Figure 5A), evidenced the formation of spherical core-shell micelles (with core radius R_core_ = 81.1 Å and shell thickness Th_Shell_ = 24.1 Å) and a relatively large aggregation numbers (N_agg_ = 144). The spherical shape was also confirmed by the near bell shape of the PPDF *p*(*r*) (Figure 5A) [71].

For the PGMA_72_-g-PEG_45_/PLA_33_ systems (Figues 5B) the SAXS data were fitted using a core–shell spherical model (with R_core_ = 64.0 Å and Th_Shell_ = 29.1 Å). In that case, despite the spherical shape being confirmed by the near bell shape of the *p*(*r*) function, relatively small aggregation numbers (N_agg_ = 3.46) were detected. Finally, the presence of a long PGMA polymer backbone favor the formation of an elongated micellar morphology for the PGMA_721_-g-PEG_45_/PLA_29_ copolymer system. The corresponding SAXS data were fitted by means of a core–shell cylindrical (form factor) model (with R_core_ = 42.5 Å and Th_Shell_ = 28.1 Å and length = 1004.1 Å), and an aggregation numbers near to the unity (Figure 5C) [71]. Generally, for an ideal rigid cylinder the *p*(*r*) is characterized by the initial bell shape at low r, followed by the inflection point, and a very linear decrease to zero at larger r [72]. In Figure 5C, the presence of another peak (shoulder) instead of a linear decay toward zero in the *p*(*r*) function may be indicative of a more flexible rod-like micelles for the PGMA_721_-g-PEG_45_/PLA_29_ diblock copolymer system.

### 3.4. Study of the Self-Assembly Processes, Phase Transitions Mechanisms and Aggregation Kinetics

Small angle scattering provides unparalleled insights into the investigation of the self-assembly processes in colloids and amphiphilic systems, as well as for the investigation of the phase transitions mechanisms and aggregation kinetics in a large variety of nanomaterial and biomaterial systems [73,74]. They also provide an accurate methodology to identify the order-disorder transition in several nanostructured material systems, including amphiphilic, polymer-based and lipid-based nanostructured systems [73,74,75,76]. A strong impact of SAXS and SANS techniques in biomaterials and nanomaterials fields of application concerns the studies of the (self-)assembly processes triggered by a variety of physico-chemical variables or (internal/external) perturbations, such as concentration (or reactants concentration), pH, ionic strength, temperature [77,78,79,80,81,82].

An important example of this type of investigation can be found in a study of the self-assembly processes and supramolecular interaction in amphiphilic systems in aqueous solutions [83,84], as well as in the case of particular class of anhydrous systems of interest for technological applications [85]. In this case, characterization by scattering techniques can highlight the reason, from the structural point of view, of the arising of a wide variety of emerging properties in amphiphile-based complex systems, from enhanced and/or anti-Arrhenian behavior of conductivity [86,87] to magnetically induced optical birefringence [88,89], from increased performances in bituminous materials [90] to the origin of the characteristic behavior of ionic liquid [91], where computational studies can support, from the theoretical point of view, experimental data [92].

**Figure 6:**
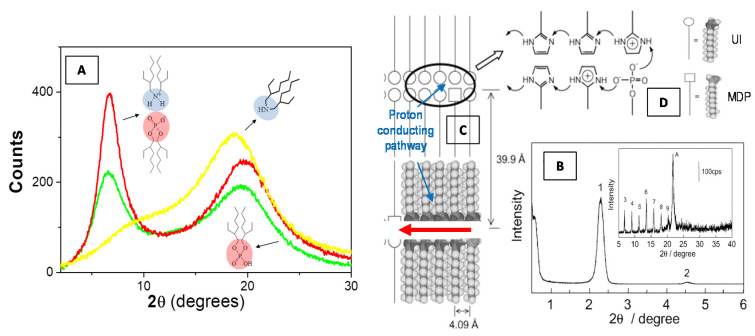
Scattering profile of a bis(2-ethylhexyl)amine/bis(2-ethylhexyl)phosphoric acid binary liquid mixture (equimolar mixture, red line) as compared to the two neat components bis(2-ethylhexyl)amine (yellow line) and bis(2-ethylhexyl)phosphoric acid (green line) (**A**). It can be noted that the mixture shows an increase of the intensity at the low-angle peak as a consequence of the local intermolecular self-assembly (**A**). X-ray diffraction pattern (peak 1) at low angle (**B**) and associated higher-order diffraction peaks at high angle (inset of **B**) regions of the 2-undecylimidzole (UI)−monododecyl phosphate (MDP) composite materials, evidence the formation of highly ordered bilayer lamellar structures [85]. The formation of a proton-conduction mechanism (**C**) is favorite by the imidazole headgroups (**D**) that are protonated by the proton transfer from neighboring MDP molecules. Adapted with permission from [85], Copyright 2019 American Chemical Society.

In Figure 6A we report SAXS profiles of a bis(2-ethylhexyl)amine/bis(2-ethylhexyl)phosphoric acid binary liquid mixture as compared to the two neat components bis(2-ethylhexyl)amine (yellow line) and bis(2-ethylhexyl)phosphoric acid (green line). The increase of the intensity at the low-angle peak evidence a local intermolecular self-assembly within the complex amphiphilic system.

Recently, Yamada and Honma [85] obtained anhydrous protonic conductivity in self-assembled (acid−base) composite material with highly ordered lamellar structures through the hybridization of acidic surfactant monododecyl phosphate (MDP) and basic surfactant 2-undecylimidzole (UI) molecules. This UI−MDP composite material has been found to form highly ordered bilayer lamellar structures (Figure 6B) (with the d spacing of 39.9 Å and a distance between alkyl chains of 4.09 Å) and to favorite a two-dimensional proton-conducting pathways (Figure 6C) within the close-packed surfactant headgroups (Figure 6D). The UI−MDP lamellar composite materials showed a high proton conductivity of 1 × 10^−3^ S·cm^−1^ at 150 °C under the anhydrous condition (with activation energy of 0.30−0.45 eV of proton conduction). These anhydrous proton-conducting membranes (without the existence of water molecules) have attracted remarkable interest for application to the polymer electrolyte membrane fuel cell (PEMFC) operated at intermediate temperature (100−200 °C) [85].

The use of the SAS techniques has also proved to be particularly important for the study of the structural properties and phase behavior of soft condensed matter and of biomolecular systems, such as proteins, lipid mesophases and (model) biomembrane systems. Particularly interesting are the time-resolved SAXS experiments for the investigation of transient processes over millisecond time range (and in a wide range of length scales), such as has the protein folding [93], the RNA folding [94] and fast conformational transitions in DNA Origami [95]. Those experiments require a very intense X-ray beam available at synchrotron radiation facility and dedicated instruments for rapid perturbation, such as a stopped flow apparatus for the fast mixing of the biomolecule with specific materials substrates, or to create a ionic strength or pH jump, or a high pressure cell for applying well-defined pressure jumps, or IR-laser irradiation to generate temperature jump (T-jump) [96,97]. For example, a new setup for time-resolved SAXS measurements using hydrostatic pressure jumps (of several hundred bars in the sub-ms regime) has been developed with the aim to investigate the fast structural changes, self-assembly, phase transitions and folding processes in various soft matter systems, including surfactants, lipids and proteins [98].

The combined SAXS/WAXS time-resolved studies were found to be extremely useful for understanding the structural features and phase behavior of lipid-based nanostructured systems.

**Figure 7:**
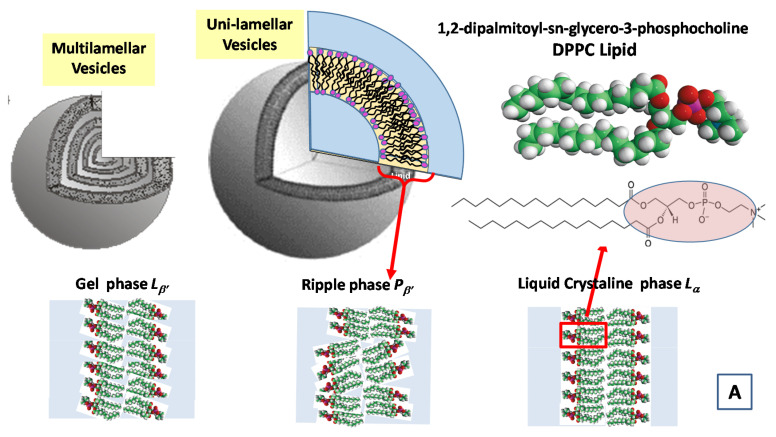

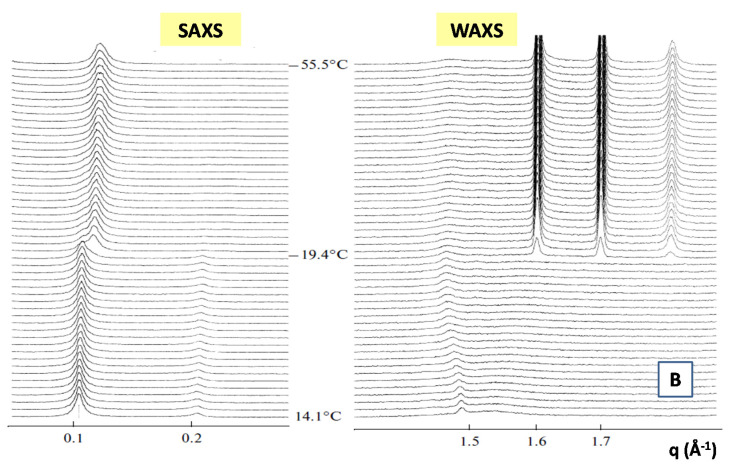
Main structural phases in water solution of a 1,2-dipalmitoyl-sn-glycero-3-phosphocholine (DPPC) lipid system (**A**). Time evolution of the SAXS and WAXS spectra of multilamellar DPPC vesicles during the cooling from temperature *T* = 14 °C to *T* = −55.5 °C (Adapted from Kiselev [99]) (**B**). The cooling rate is 1.5 °C/min and data acquisition time is 1 min for each spectrum.

In Figure 7, we report the time evolution of the SAXS and WAXS spectra during the cooling of multilamellar DPPC vesicles in excess water (from temperature *T* = 14 °C to *T* = −55.5 °C) [99].

The SAXS spectra in the temperature range from *T* = 14.1 to *T* = −19.4 °C evidence the beginning of the gel L_β’_ phase and the transition toward the crystallization to the L_c_ phase (See Figure 7A). A shift of the diffraction peak at *T* = −19.4 °C to the region of larger values of the scattering vector q evidences the decrease of the membrane repeat distance d by Δd = 5.9 Å, which is connected to the ice formation in the bulk water surrounding the vesicles. The WAXS spectrum at *T* = −19.4 °C evidences three diffraction peaks corresponding to hexagonal lattice of ice crystals with minimal interfacial distances of 3.9 Å, 3.6 Å and 3.4 Å respectively. At *T* = 14.1 °C, the WAXS spectra from hydrocarbon tails evidences the (2,0) two diffraction peak at q = 1.49 Å^−1^ and the (1,2) wide “shoulder” at q = 1.53 Å^−1^, which correspond to the rectangular cell (with the lattice constants at = 8.46 and b = 9.38 Å), of the package of DPPC hydrocarbon tails in the gel L_β’_ phase. The peaks (2,0) and (2,1) are monotonously moving away from each other during the cooling down to the temperature of ice formation reaching the positions 1.46 Å^−1^ and 1.58 Å^−1^, which corresponds to the rectangular cell (with lattice constants a = 8.61 Å and b = 8.97 Å). Those trends evidence the slow transition of the L_β’_ phase toward the crystalline L_c_ phase [99].

It is worth pointing out that SAS techniques offer unique structural information, from a higher resolution viewpoint and over several length scales, of both the atomic and (macro-)molecular organization in complex nanostructured systems that are characterized by a hierarchical supramolecular structure. This holds particularly true in the case for many porous nanomaterials, which exhibit micro-, meso-pores hierarchical structures (even with a secondary nanomaterial or compounds confined within the pores), such as fuel-cell and battery materials, as well as in supported catalysts [100,101,102,103]. SAXS and SANS techniques are among the few experimental approaches that can be used for the in situ study of the synthesis and self-assembly processes occurring (at the mesoscopic scale) in nanoporous and mesoporous materials. They also provide an advanced understanding of the main factors and driving interactions that lead to the morphologies and structural organization of those materials, which, in turn, give rise to their peculiar properties and functions [100,101,102,103]. More specifically, the hybrid organic–inorganic materials obtained through solution precipitation using sol–gel chemistry have been more and more investigated in the past decades, because of their large applications in the various field of material science and biotechnology [104,105,106]. Although the comprehension of the mechanism of formation followed by these complex nanomaterials is difficult, they are, undoubtedly, of fundamental importance for the control of the material structure and its properties. Recent SAS studies evidenced the great versatility of porous materials for the development of advanced nanoplatforms (because of the rich and combined presence of a variety of surface end-groups and large surface areas), e.g., by the tuning of the hydrophilic/hydrophobic nature of the surface, incorporation of metal complexing, luminescent hosts, mechanical and electronic properties [105,106,107].

Recently, the self-assembly of surfactant-templated mesostructured (organically modified) silica nanostructures was investigated by means of an in situ time-resolved SAXS experiment [106]. In that case, the use of intense synchrotron radiation X-rays source [63], with a quick temporal resolution (of seconds), in combination with suitable modeling of micelles evolution allowed to elucidate the mechanisms of formation of the organically modified (phenyl, vinyl and methyl) silica materials with periodic meso-structures, obtained with the use of the cetyltrimethylammonium bromide (CTAB) micellar aggregates template [106].

**Figure 8:**
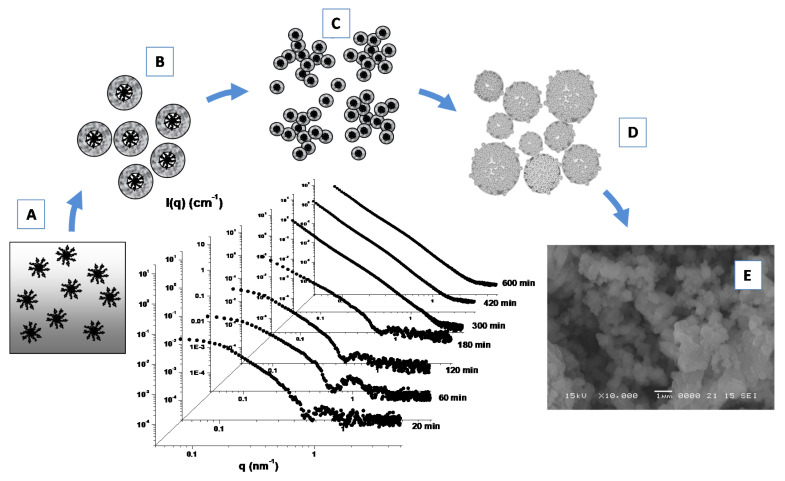
SAXS time evolution and formation of porous Linde type A (LTA) zeolite nanostructures from a clear sol containing amphiphilic PDMS-b-PEO block copolymers micelles as templating agents (**A**). Incorporation of LTA aluminosilicate components onto the micellar surface favorites the formation of core-shell primary units (**B**), that aggregate and lead to the formation of extended secondary units (**C**). Further fusion and rearrangement of the secondary units leads to the formation of final submicrometer aggregates (**D**), as confirmed by SEM experiments (**E**). Adapted with permission from Lombardo et al. [107], Copyright 2013 American Chemical Society.

Furthermore, the formation of porous Linde type A (LTA) zeolite nanostructures from a clear sol containing amphiphilic PDMS-b-PEO block copolymers micelles (Figure 8A) as templating agents has been investigated by means of SAXS experiments (Figure 8) [107]. During the early stage of the growth process the formation of core-shell primary units (of 4.8 nm) with a core-shell morphology (Figure 8B) was driven by the zeolite aluminosilicate components incorporation onto the micellar surface. A successive (and progressive) aggregation process among these primary units leads to the formation of extended secondary units (Figure 8C), which undergo a further cross-linking, fusion and rearrangement, thus, leading to the formation of final submicrometer aggregates (Figure 8D), as confirmed by SEM experiments (Figure 8E). Those studies furnish important insights for the understanding of the self-assembly processes involved in the design and development of hybrid organic–inorganic mesoporous materials, as well as stimulating the study of alternative methods for the production of novel materials with new characteristics and properties.

### 3.5. Scattering from Fractal Aggregate: Porod Limit

Many aggregation processes in material science and nanotechnology lead to the formation of fractal structures, possessing self-similar characteristic in a given spatial interval, whereby the mass *M* in a volume *R^Df^* follows the power law *M* ~*R^Df^* (where *D_f_* is called the fractal dimension). The fractal dimension defines the scaling relationship between mass (or particle number) and the enclosed volume, and may furnishe an (indirect) indication of the physical mechanism of formation during particular aggregation processes [108]. Small-angle scattering can be used to study the geometry of aggregates of colloidal particles in solutions, because it provides a direct measure of the fractal dimension. The fractal dimension of a particle can be determined analyzing the power-law regime of the scattered intensity *I*(*q*) ~*q*^−*α*^ [108], where the exponent *α* is related to the fractal dimension *D_f_* of the scattering structures. For fractals with a finite cutoff correlation length *ξ*, the SAS intensity profile obeys the following scaling law (where *ξ* is the correlation length) [108].

(7) $$P\left(q\right)=\frac{sin\left[\left(D_{f}-1\right)arctan\left(q\xi \right)\right]}{\left(D_{f}-1\right)q\xi {\left(1+q^{2}\xi^{2}\right)}^{{\left(D_{f}-1\right)}^{2}}}$$

For a mass fractal, it is possible to show *α_m_* = *D_m_* and 1 < *α_m_* < 3 in a three-dimensional space. In contrast, we have *α*_s_ = (6 − *D_s_*) for surface fractals (with surface fractals exponents between 3 < *α*_s_ < 4). If *D_s_* = 2, we obtain the well-known Porod’s law *I*(*q*) ∝ q^−4^ for non-fractal structures with smooth interfaces.

**Figure 9:**
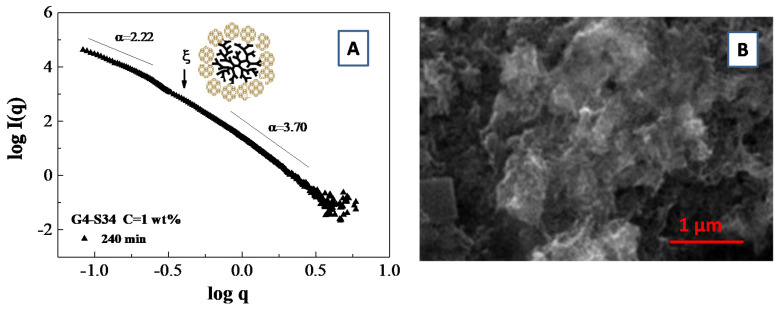
SAXS fractal analysis for a system of Silicoaluminophosphate SAPO-34 zeolite nanoparticle on template of polyamidoamine (PAMAM) dendrimer (generation G = 4.0) at the water concentration of C = 1 wt.%. The log–log plot of the SAXS intensity profile during the late stages of SAPO-34 zeolite synthesis identify the linear regions for the fractal analysis (**A**). Scanning electron microscopy (SEM) images of the aggregates generated during dendrimer template directed SAPO-34 zeolite synthesis (**B**).

An example of a fractal analysis obtained from small angle scattering data is presented in Figure 9A, where we report the slope of log*I*(*q*) vs. log(*q*) for a system composed of polymamidoamine (PAMAM) dendrimers templated silicoaluminophosphate SAPO-34 zeolite nanoparticle which undergoes an aggregation process [103]. More specifically, the fit of the SAXS curves furnish an average slope of *α* = 2.22 (i.e., a mass fractal dimension of *D_m_* = 2.22) in the low q region and an average slope of *α* = 3.70, corresponding to a surface fractal dimensions of *D_f_* = 2.30 in the large q region. Those results furnish an indirect indication of the presence of porous aggregates, which were confirmed by scanning electron microscopy (SEM) images of the aggregates (Figure 9B)

It is important to highlight that the wavevector range for the mass fractal aggregates (i.e., the range exhibiting a straight line in SAXS spectra) is limited from below by the size of the building units (that corresponds to the shell radius R_2_ detected during the early stage of the synthesis process), that defines the lower cutoff value of *ξ* = 2π/R_2_ (which is indicated by an arrow in Figure 9A). On the other hand, the upper cutoff value of *ξ_U_* = 2π/D_aggr_ (which delimits the range of the fractal linear behavior) is defined by the total aggregate size D_aggr_. In our specific case, the upper cutoff value of *ξ_U_* is delimited by the value of the lower accessible wavevector q_0_ by the SAXS experiment [103].

### 3.6. Analysis of the Structure Factor S(q): Study of the Interpaticles Interaction

At the most concentrated solutions, SAS furnishes also interesting information about the effective interparticles interaction potential. The structure factor *S*(*q*) for a dispersed system of interacting particles can be written as [109,110]:

(8) $$S(q)=1+\int_{0}^{\infty }4\pi^{2}\rho_{C}\left[g(r)-1\right]\frac{\sin(qr)}{\left(qr\right)}dr$$

where *ρ_C_* = *c/M* is the particle number density (number of particles per unit volume). This last relation provides a way to connect the structure factor *S*(*q*) with the radial pair correlation function *g*(*r*) (i.e., the probability that two particles stay at distance *r* in the system). Equation (8) can be solved numerically in the framework of the Ornstein–Zernike integral equation (OZ) for the total correlation function *h*(*r*) [110,111]. In the past decades, different calculation protocols, which make use of the so called “closure relations”, have been proposed for the solution of the OZ equation, including mean spherical approximation (MSA), Percus–Yevic (P–Y), hypernetted chain (HNC), Rogers–Young (RY) closure relations [109,110,111]. These calculation protocols request the modelling of the effective pair inter-particles potential *U*(*r*) through the choice of the relevant structural parameters of the system (such as particles size, concentration, effective surface charge, ionic strength) [26]. The most investigated form for the inter-particles potential *U*(*r*) in the field of nanotechnology is certainly the so called DLVO potential, proposed by Derjaguin–Landau–Verwey–Overbeek in a pioneering work describing the stability of colloids [109].

According to this model the interaction potential between two identical nanoparticle (i.e., spherical macroions of diameter *σ* = 2R) placed at a (center to center) distance r is determined by the balance between the van der Waals attractive forces *V_A_* and the screened Coulomb (repulsive) interaction *V_R_*, and is described by the following equations:

(9) $$V_{A}=-\frac{A}{12}\left[\frac{1}{r^{2}-1}+\frac{1}{r^{2}}+2\ln\left(1-\frac{1}{r^{2}}\right)\right]$$

(10) $$V_{R}=\frac{Z_{0}e^{2}}{4\pi \varepsilon {\left(1+\kappa \sigma \right)}^{2}}\frac{e^{-\kappa (r-\sigma )}}{r}$$

where *A* is the Hamaker constant, *e* is the unit of electron charge, *K_B_* the Botzmann constant, *N_a_* the Avogadro number and *κ* = (λ_DH_)^−1^ = (8π*e*^2^*N_a_I*/*εK_B_T*∙10^3^)^1/2^ is the Debye–Huckel screening length, which is determined, at a given temperature T, by the ionic strength I of the solvent (in mol/L).

**Figure 10:**
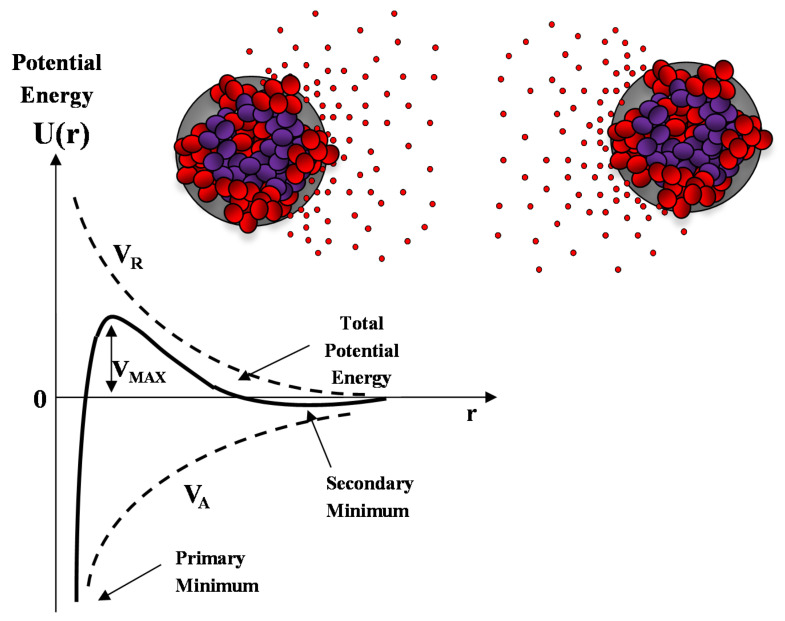
Sketch of the DLVO interaction potential energy as a function of particle separation r. The net potential energy is given by the sum of the double layer repulsion *V_R_* and the van der Waals attractive forces *V_A_*.

A sketch of the Derjaguin-Landau-Verwey-Overbeek (DLVO) interaction energy as a function of the nanoparticle separation is reported in Figure 10. The presence of an energy barrier resulting from the repulsive force prevents two particles adhering together while approaching one another. Particles with a sufficiently high repulsion can avoid flocculation and the colloidal system will be stable. On the other hand, particles colliding with sufficient energy are able to overcome that barrier, thus, favoring an aggregation process between particles. In certain solution conditions (e.g., at high ionic strength), there is a possibility of a secondary minimum, where a weaker and reversible adhesion between particles may exist. These weak aggregates, although sufficiently stable, may dissociate during Brownian motion, or under an externally applied perturbation (such as a temperature increase).

**Figure 11:**
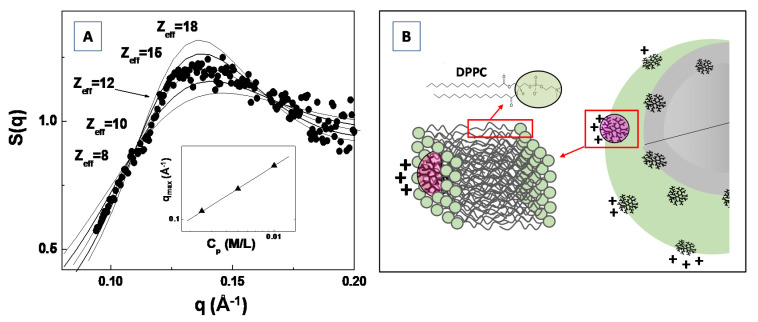
Analysis of the SAXS structure factor *S*(*q*), by means of the theoretical hypernetted chain (HNC) closure relation, for the amine terminated generation G4.0 PAMAM dendrimers in water solution (**A**). Charge attenuation effect of G4.0 PAMAM dendrimers, during their inclusion in model DPPC lipid bilayer system (**B**).

In Figure 11A, the experimental SAXS structure factor *S*(*q*) for a system composed of generation G4.0 polyamidoamine (PAMAM) dendrimers in water solution (at the concentration of c = 1.4 mM) is compared with the structure factor calculated by modeling the (long range) inter-dendrimers interaction potential with a screened Coulomb (repulsive) interaction. The Figure shows that the adopted model for the inter-particles interaction potential (and assuming the hypernetted chain closure relation [109,111]) satisfactorily reproduces the experimental results, by assuming an average dendrimer effective charge of Z_eff_ = 12 (in unit of electron charge |e|). The effective dendrimers charge plays an important role in nanostructures self-assembly processes as the electrostatic interaction represents an important control factor in many smart applications in bio-nanotechnology [112,113,114].

Recently, Lombardo et al. [115] investigated the effect of the inclusion of dendrimers in model lipid DPPC membranes by means of SAXS, Zeta-potential, Raman and dynamic light scattering experiments. They evidenced a considerably attenuated dendrimer charge of Z*_eff_ = 1.50, for G4.0 PAMAM dendrimers embedded in DPPC lipid vesicles, if compared with the dendrimer effective charges of Z_eff_ = 12.0 previously obtained by SAXS analysis of structure factor *S*(*q*) (Figure 11A). The difference in the detected dendrimer charge has been explained by assuming that dendrimers are embedded (more or less deeply) within the bilayer hydrophobic region (as depicted in Figure 11B). Moreover, an increase in PAMAM concentration above a given threshold (which depends on the dendrimers molecular weight) causes a perturbation of the bilayer structure, and is characterized by an instability of the liposome colloidal stability [115]. The findings of this study allow to rationalize the effect of nanoparticles interaction with lipid vesicles (and biomembranes in general), as well as to provide important insights about the perturbation of lipid bilayers membrane induced by nanoparticles inclusion. The study of the inter-nanoparticle interaction (by means of SAS experiments) is a fundamental step for the control of the aggregation tendencies of complex bio-systems in solution, and may stimulate new approaches for the study of biological processes involved in biomaterials systems.

Recently, an increasing number of investigation evidences the use of the scattering methods in conjunction with molecular dynamics simulation approaches (so called “scattering-guided molecular dynamics simulations”), for elucidating the main structural properties of biomolecules and biomaterials [116,117]. For example, the combination of coarse-grained molecular dynamics simulations and SAXS experiments have been used to efficiently investigate protein and nucleic acids structure-dynamics properties, by using high-resolution structural data of scattering factors [116]. Moreover, molecular dynamic (MD) simulations have been useful in revealing detailed molecular features within a lipid bilayer, by reproducing small angle X-ray data [118]. Furthermore, the small angle scattering form factors of a lipid bilayers system composed of 1-palmitoyl-2-oleoyl-sn-glycero-3-phosphatidylserine (POPS) lipid were simulated from MD trajectories through the calculation of the number density distributions of all atoms [119]. More specifically, neutron or X-ray form factor was simulated by means of the Fourier transform of the solvent-subtracted neutron scattering length density (NSLD) and electron density (ED) profiles [119]. The direct comparison between the experimental form factors and MD simulation allows to choose the bilayer system that best agrees with the experimental data (best simulated bilayer). Finally, the synergic combination of neutron scattering and fully atomistic molecular dynamics simulations has proven to be an useful tool to investigate structural and dynamical features of linear homopolymer melts at different size scales regions (from the inter-molecular, molecular and even large-scales), including structural relaxation processes, local short-range order and motions [120]. The increasing use of those combined approaches is of relevant importance, in order to elucidate the processes arising in biomolecules, polymer and material systems with increasing complexity, including soft matter field and biomaterials.

### 3.7. SAS Investigation of Structure-Function Relationship in Biomolecules and Biomaterials

In this section, we show how the small angle scattering techniques can contribute substantially to the understanding of the relationship between nanoscale structure of biomatererials (and biomolecules) and the relevant interactions, which finally determine their complex functions in biological systems. Those investigations furnish new insights that facilitate the identification of the relevant parameters for the control of the self-assembly properties and the final microstructure, in view of possible biomedical and biotechnology applications.

#### 3.7.1. Drug–Biomembranes Interaction

SAS techniques have given substantial contribution to the investigation of the structural organization of nanocarriers composed of polymer-based and lipid-based micelles, polymersomes and liposomes, (hybrid) organic/inorganic nanoparticles and their interaction with biomaterials or bioactive macro-molecules [121,122,123,124,125,126]. More specifically, SAS makes it possible to deeply investigate the structure of drug nanocarriers alone or loaded with active drugs in their aqueous environment. Structural parameters (such as dimensions, shape) and electron density maps obtained by high resolution SAS investigations help to localize the active guest molecules in self-assembled nanostructures, and to elucidate the effect of drug inclusion on their colloidal stability [121,122,123,124,125,126].

Recently, the combination of SAXS technique with a remote controlled addition of synovial fluid (or buffer containing 2% *w/v* albumin), allowed for the investigation of the hydration-triggered structural transition, during the exposure of a lipid precursor (consisting of monoolein/castor oil lipid mixture) to excess synovial fluid (or excess buffer) [125]. The use of time resolved synchrotron SAXS experiments evidenced a fast generation (within few seconds) of inverse bicontinuous cubic phases (as a function of the lipid composition and the albumin content). This study enhances the interest in the design of injectable (low-viscous) stimulus-responsive lipid precursors that convert to inverse non-lamellar liquid crystalline phases (in situ, at the administration site in the body), thus, stimulating the investigation of sustained (intra-articular) drug release applications [125]. In a study of Giulimondi et al. [126], high-resolution SAXS experiments allowed to evaluate the impact of protein adsorption on the inner structure and the colloidal stability of different model nanocarriers systems composed of cationic 1,2-dioleoyl-3-trimethylammonium-propane (DOTAP), neutral dioleoylphosphocholine (DOPC) and anionic 1,2-dioleoyl-sn-glycero-3-phospho-(1′-rac-glycerol) (DOPG) [126]. Interestingly, the exposure of DOTAP lipid nanocarriers to low plasma concentration induced the formation of multilamellar structure as demonstrated by the presence of Bragg peaks. More specifically, the plasma proteins were able to coat the positively charged surface of DOTAP liposomes, thus leading to the formation of (protein-decorated) vesicles aggregates. The successive stresses generated by (protein-mediated) liposome–liposome adhesion caused a local rupture of vesicles, thus, leading to the formation of multilamellar nanostructure. The authors evidenced that the formation of clusters can start only when plasma proteins completely coat the external surface of liposomes. However, no plain evidence of multilamellar structure was found at high plasma concentration. In this last case, it was suggested that the massive protein binding to liposome surface could create an electrostatic repulsive barrier between liposomes, as evidenced by zeta-potential results [126]. Those results confirm the important role of charge effects in the liposome-nanocarriers interaction, and its special role in the preservation of the colloidal stability of bio-nanocarrier systems [127,128,129]. Furthermore, those results demonstrate that the protein corona plays a special role during interaction of immune cells with liposomes systems. The study also evidenced that the pre-coating liposomes with an artificial corona made of human plasma proteins sensitively decreases capture by leukocytes in whole blood [126]. Those results stimulate the development of efficient strategies to overcome the sequestration of nanocarriers by the immune cells, thus, enabling their prolonged circulation in vivo.

In a recent high-resolution synchrotron SAXS investigation it has been shown that the M2 protein from the influenza A virus is topologically active for the generation of negative Gaussian curvature (NGC) in lipid membrane, and necessary to the influenza budding and scission [130]. More specifically, the M2 protein is able to restructure lipid membranes into bicontinuous cubic phases that are rich in NGC, while the mutations to the protein helix (which reduce its amphiphilicity) diminish budding and attenuate the NGC generation [130]. This knowledge provides important insights for the design and engineering of specific budding proteins and suggests the development of therapeutic strategies for the next generation of antiflu drugs and chemotherapies.

The NGC re-structuring enables various types of membrane permeation processes. Recently, an SAXS investigation evidenced that antimicrobial peptides (AMPs) preferentially generate Gaussian membrane curvature in model bacterial cell membranes [131]. Short cationic, (amphipathic) antimicrobial peptides (AMP), are multifunctional molecules that selectively permeate microbial membranes, and play a special role in host defense as direct microbicides and modulators of the immune response. From in vitro studies, amphipathic AMPs are inferred to selectively target and disrupt microbial membranes through a combination of non-specific electrostatic and hydrophobic interactions, thus, leading to membrane permeabilization, depolarization, leakage and eventual cell death [131,132]. More specifically, the interaction between the anionic membrane and the cationic AMPs leads to strong electrostatic attraction that favors the binding of the AMP to the membrane. Upon adsorption to the membrane, the hydrophobic portions of the AMP insert into the non-polar region of the bilayer, thus, leading to membrane destabilization and loss of barrier function [131,132].

It is worth pointing that the multicomponent character, as well as the specific nanostructure of both drug nanocarriers and biomembranes, strongly influences the intracellular interaction and the induced structural transition (such as rupture or vesicle aggregation), thus, influencing in a sensitive way the drug bioavailability [133,134]. In this respect, the SAS structural investigation of multi-components drug-biomembranes (or even drug-live cells) is a complex task, due to the need for extensive modelling of the scattering contributions from all (or the main) sample components (e.g., lipids, proteins, carbohydrates, DNA). Hence, this complex compositional modelling often needs complementary information coming from other complementary techniques [135]. The large number of involved parameters requires novel approaches based on advanced global optimization methods, such as statistical data evaluation schemes, machine learning methods or genetic algorithms [136,137].

#### 3.7.2. Conformational Changes in Flexible Biomolecules: Ensemble Fitting of the SAS Data

SAS are well established methods for the study the structure and dynamic transitions of biological macromolecules in solution. They offer fundamental information about (macro-) and bio-molecular aggregation and assembly states, folding, unfolding, shape conformation in solution [138,139,140,141]. Particularly interesting is the use of SAS methods for the study of equilibrium mixtures or non-equilibrium processes, like protein folding/unfolding or (dis-)assembly pathways. In those cases, the SAS data treatment and interpretation are typically restricted to overall parameters (such as dimensions connected to sphere/cylinder shapes, radius of gyration Rg). This analysis yields limited information about the kinetics of possible intermediate processes, (i.e., folding/unfolding intermediates, aggregation).

One of the major versatility of SAS techniques lies in the fact that they are applicable not only to monodisperse solutions of relatively rigid and well-folded macromolecules, but also to complex mixtures of different types of biomolecular systems that have conformational flexibility and polydispersity, such as disordered (macro-)molecules, oligomeric mixtures, RNA folding, multi-domain proteins (MDPs) or intrinsically disordered proteins (IDPs) [138,139]. The complex (structure-dynamic) behavior of specific ensembles of bio-macromolecules mediates essential processes in biology. For this reason, the study of the main mechanisms that drive the molecular interactions (and functions) of flexible (bio-)molecules could benefit from using alternative approaches, such as those traditionally employed in structural biology [138,139,140]. In this respect, the use of specific multi-scale modeling and dynamic molecular machines approaches make it possible to create macromolecules images and configurations for modeling supramolecular complexes, allosteric mechanisms, in diverse processes ranging from eukaryotic DNA replication processes, recombination/repair to biomembrane, and self-assembly systems [138,139,140].

Bernadò et al. [141] developed an ensemble optimization method (EOM), which represents the first approach that introduces the concept of ensemble fitting of the SAS data from multiple protein conformations in solution, and provides insights into structural features and quantitative information about flexibility [141]. The suitable combination of SAS methods with complementary experimental approaches (such as NMR, X-ray crystallography) and computational methods make it possible to characterize the quaternary structure of complexes (formed of biological macromolecules), starting with the (high resolution) rigid-body models of the individual subunits [140,141]. Thanks to modern synchrotron radiation facility, it is possible to develop high-throughput SAXS (HT-SAXS) methods based on fast data collection (with times acquisition within seconds), robotic sample changers, automated data collection and analysis pipelines [142,143]. This approach represents a useful tool within the high-throughput screening pipeline of modern drug discovery, and makes it possible to identify oligomeric states, shapes/structures transition and aggregated/unfolded conformations of proteins. Within this approach, the ultrastructural alterations caused by protein synthesis inhibitors, RNA polymerase inhibitors and membrane disruptive antibiotics can be investigated. More specifically, the SAXS structural characterization is able to describe the level of degradation of the cell wall by analyzing the morphological patterns. While conventional tests to identify the mode of action of an antimicrobial substance require days or weeks per single substance, with the HT-SAXS approach it is possible to speed up the process by integrating the SAXS method into a drug research pipelines, and obtain ultrastructural information of large sets of unexplored substances. Moreover, in contrast to real space imaging techniques, SAXS makes it possible to obtain nanoscale information averaged over approximately one million cells [141,142,143].

Recently, a high throughput SAXS method allowed to classify the mode of action for novel antimicrobial drug (antimicrobial peptides) providing ultrastructural information averaged over approximately one million *E. coli* cells in high throughput experiments (with 1 s measurement, 1 min sample changing, 20 μL sample volume) [143]. The study evidenced that an antimicrobial peptide with unknown mode of action could be distinguished from all other antibiotics tested, as it exhibited a different mode of action [143]. This approach is, therefore, an attractive candidate for drug development against multi-drug resistant bacteria.

#### 3.7.3. SAS Structural Investigation of Bio-Polymers and Hydrogels

Structural information at the nanoscale obtained from SAS methods could be used to understand and explain the mechanical properties of biopolymer-based nanomaterials. Very often, starting from the component particles, biomaterials self-assemble into fiber morphology, which is characterized by a significant polydispersity [144]. Both SANS and SAXS techniques are able to furnish useful information about the average radius of gyration of the fiber cross-section (*Rc*), its mass-fractal dimensions (*D_f_*) (which describes the building blocks organization within the morphology of the network), the correlation length (*Lc*) (which indicates the mesh size network) and the persistence length of the fiber (*Lp*) (which is connected to the mechanical properties of biomaterials) [145,146,147].

Using combined SAXS and SANS techniques, Taraban et al. were able to explain the mechanical advantage of homo-chirality in peptide hydrogels, by exploiting the complementarity of the two techniques [148]. More specifically, due to the high flux of an X-ray beam from a synchrotron, they were able to reduce the SAXS data collection time down to 0.2 s, which facilitated the real-time monitoring of the gelation process. Finally, high resolution SAS makes it possible to investigate mechanical properties of biopolymer-based biomaterials by tuning various combinations of structurally different components. For instance, it has been shown that the engineering of peptide-based biomaterials by the addition of less stiff, but more elastic, polysaccharide networks increases the mesh size of the network, thus, resulting in a more flexible biomaterial that is less prone to breaking [149,150]. Those hybrid biomaterials are stiffer than polysaccharide networks alone and present enhanced mechanical properties in comparison to (brittle) pure peptide networks.

## 4. Light Scattering Techniques

In the last few decades, light scattering methods have been widely used to study colloidal, polymer and biomolecular systems (including protein, lipids, oligonucleotides, carbohydrates) in a wide range of environments and solution conditions [151,152]. Light scattering techniques yield information about the molecular weight, size, structure and dynamics of a large variety of material system (sample). When a monochromatic beam of (laser) light encounters a system consisting of macromolecular objects in solution, it scatters the light in all directions in a manner that depends on the size/shape and concentration of the macromolecules. In a static light scattering experiment, the scattered intensity of light is detected (as time-averaged quantity) as a function of scattering wavevector q, while its analysis provides important information on molecular weight, size and shape of macromolecules. On the other hand, the Brownian motion of macromolecules in solution causes an intensity fluctuation of the scattered light. Analysis of the diffusion coefficient (*D*) can be related with the hydrodynamic dimension of macromolecular system under investigation, and can be obtained by the dynamic light scattering method. In the following section, we analyze the two techniques, with a special focus on their application for the characterization of bio-nanomaterials.

### 4.1. Static Light Scattering (SLS) and Small Angle Light Scattering (SALS)

In a static light scattering (SLS) experiment, we can measure a variety of material system properties, including molecular weight, shape and dimensions (in the range between 10–1000 nm) and crucial parameters connected to nanoparticles interactions (such as the second virial coefficient *B*_2_). It is worth pointing out that the material (and macromolecular) system under investigation should have a refractive index that is different from that of the dispersing medium (solvent). Moreover, it should be transparent (non-turbid) and should not absorb light (at the wavelength of operation).

**Figure 12:**
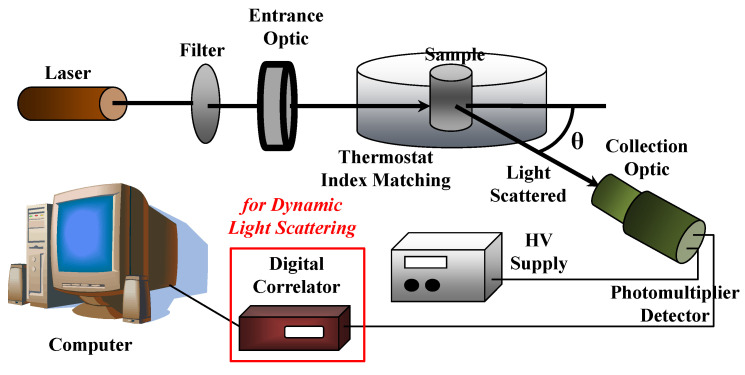
Sketch of a (static and dynamic) light scattering setup.

A sketch of a typical (static and dynamic) light scattering set up is reported in Figure 12. A high intensity monochromatic light of a laser beam impinges in a solution containing the sample under investigation, which is immersed in a temperature-controlled index matching system (thermostat bath), while a detection system will measure the scattering intensity as a function of the scattering wavevector q (i.e., at different scattering angles θ) by using, for example, a fast photon detector, such as a phototube or a photodiode. As we will describe later, the insertion of a digital correlator allows to perform photon correlation spectroscopy measurements in dynamic light scattering experiments.

In SLS experiments, the scattering intensity *I*(*q*) is measured as a function of scattering wavevector *q* = (4π*n*_0_/λ_0_)sin(θ/2), where *n*_0_ is the refractive index of the solvent (*n*_0_ = 1.33 for water at *T* = 20 °C). SLS measurements are performed with variable-angle detection system, obtained by either rotating around the sample one detector on a goniometer arm or fixing several detectors at different scattering angles. For example, assuming an HeNe laser operating at wavelength λ_0_ = 632.8 nm, the scattering angles θ, which vary from 30° to 135°, provide a q-range from 7 × 10^−4^ Å^−1^ to 2.4 × 10^−3^ Å^−1^. The corrections to the absolute scattering intensities *I*(*q*) is performed by using toluene as sample reference (for which the excess Rayleigh ratio is known *R_toluen_* = 1.3522 × 10^−5^ cm^−1^ at λ = 632.8 nm) and is written as a function of the sample scattering intensity I, the solvent scattering intensity *I*_0_, and the toluene refractive index (*n_toluene_* = 1.49, at 20 °C)

(11) $$R\left(q\right)=\frac{I-I_{0}}{I_{Toluene}}{\left(\frac{n_{0}}{n_{Toluene}}\right)}^{2}R_{Toluene}\left(q\right)$$

The absolute excess scattered intensity *R*(*q*), can be expressed as a function of the normalized form factor *P*(*q*) and the structure factor *S*(*q*) [151,152,153]:

(12) $$R\left(q\right)=KM_{W}cP\left(q\right)S\left(q\right)$$

where *K* = (4π^2^*n*^2^/λ_0_^4^NA)·(*dn/dc*)^2^ is the optical constant, *M_w_* the molecular weight of the scattering objects, *c* the mass concentration, *n* the refractive index of the solution, λ_0_ is the wavelength of light in vacuum, *N_A_* the Avogadro number and *dn/dc* the refractive index increment with concentration (which represent the optical contrast between the solvent and solute molecules). The *dn/dc* value can be determined by measuring the refractive index of solutions at different concentrations of the solute molecules (or with table values).

Useful information about the scattering objects molecular weight *M_w_* can be obtained from the laser light scattered intensity at scattering angle θ = 90° through the virial expansion. At low concentrations, in fact, the structure factor can be approximated (virial expansion) as *S*(*q*) = 1/(1 + 2*B*_2_*·c*) (where *B*_2_ is the second virial coefficient). Then, for low concentration c and for low wavevector q we obtain the following relation for the Rayleigh ratio (*Kc/R*) *=* (1/*M_W_*)·[1 + (*R_g_*^2^*q*^2^)/3]·(1 + *2B*_2_) [153]. Moreover, at the scattering angle θ = 90° (i.e when *q* → 0), we can assume that *P*(*q*) ≈ 1 [153], and substitution of *S*(*q*) and *P*(*q*) into Equation (11) furnishes absolute excess scattered intensity.

(13) $$\frac{Kc}{R_{90}}=\frac{1}{M_{W}}+2B_{2}c$$

In general, the measurement of the absolute excess scattered intensity *Kc*/*R*_90_ (at the scattering angle of θ = 90°) as a function of copolymer concentration *c*, makes it possible to obtain the molar mass *M_W_* of nanoparticles from the intercept, and the second virial coefficient *B*_2_ from the slope. Useful information about the particle molecular weight *M_W_* (and of aggregation number N_agg_, in the case of aggregates) can be obtained from the laser light scattered intensity at scattering angle θ = 90° through the virial expansion (corrected from the CMC contribution) [152,153].

(14) $$\frac{K\left(c-c_{CMC}\right)}{R^{90*}-R_{CMC}^{90{}^{\circ}}}=\frac{1}{M_{W}}+2B_{2}\left(c-c_{CMC}\right)$$

**Figure 13:**
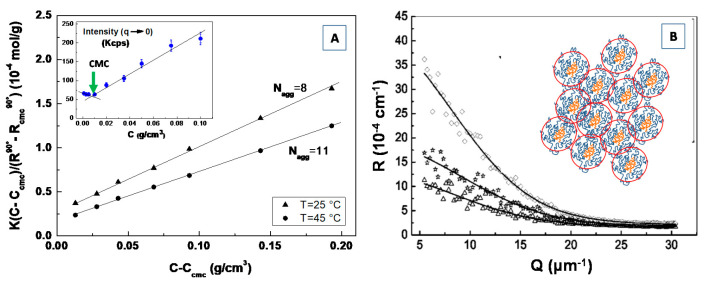
Analysis of the SLS for the water solution of PDMS-b-PEO block copolymer. Normalized inverse absolute intensity of micelles as a function of copolymer concentration at the two temperatures *T* = 25 °C (triangles) And *T* = 45 °C (squares), and the corresponding calculated mean aggregation number N_agg_ of the micelles (**A**). Excess absolute intensity profile at *T* = 25 °C at three different concentration: c = 0.05 g/cm^3^ (up triangles), c = 0.1 g/cm^3^ (stars), and c = 0.2 g/cm^3^ (diamonds) (**B**). The continuous lines are the fit according to the polydisperse sphere form factor.

In Figure 13A, we report the Debye plot as a function of the concentration c for an aqueous solution of PDMS-b–PEO diblock copolymer (with *M_w_* = 5000, composed of 82 wt% of PEO) at two different temperatures of *T* = 25 °C and *T* = 45 °C. From the linear fit, we estimated the aggregates molecular weight, and (by dividing by the diblock *M_w_* = 5000) we obtained the micelles average aggregation number of N_agg_ = 8 (at *T* = 25 °C) and N_agg_ = 11 (at *T* = 45 °C). In the inset of Figure 13A, the light scattering intensity, extrapolated to the zero scattering angle (*q* → 0), started to increase (with increasing concentration) at the concentration of *c* = 0.007 g/cm^3^ and identified the onset of the micellization process (critical micellar concentration CMC).

In Figure 13B, we report the excess absolute intensity profile (at *T* = 25 °C) for the system composed of an aqueous solution of PDMS-b–PEO diblock copolymer, at three different concentrations: *c* = 0.05 g/cm^3^ (up triangles), *c* = 0.1 g/cm^3^ (stars) and *c* = 0.2 g/cm^3^ (diamonds) (B) [154]. The continuous lines are the fits according to the polydisperse sphere (with a polydispersity factor Δ), *R*(*q*) = *R*(0)∫[*F*(Δ)]^2^*d*(Δ) + *X_B_*, where the term *X_B_* represents the contribution of micelles to the total scattered intensity.

(15) $$F\left(\Delta \right)=\frac{3\left\{sin\left[q\left(R_{C}+\Delta \right)-q\left(R_{C}+\Delta \right)cos\left[q\left(R_{C}+\Delta \right)\right]\right]\right\}}{{\left[q\left(R_{C}+\Delta \right)\right]}^{3}}$$

The fit furnishes radius of aggregates of *R_c_* = 1800 ± 300 Å (which has been found to be independent from concentration and temperature variations).

Useful structural characterization of bio-nanomaterials can be obtained by the use of small angle light scattering (SALS) [155,156,157]. The SALS apparatus consists of a monochromatic light source (e.g., a He/Ne laser) directed through the sample, while a charge-coupled device (CCD) camera detector will collect the scattering image (in the form of concentric rings) with a wide scattering vector range [155,156]. The intensity detected by the CCD, which differentiates intensity variances with different colors, gives size information of micro-particles present in the sample. The SALS setup is particularly suited for the investigation of material (dispersed) systems with a weak scattering power and/or a time-dependent structure evolution in a wide spatial range (with sub-micrometer resolution). An indication of the sample anisotropy can be detected by the presence of spots in the detected image. It is worth noticing that, while the theory behind SALS is the same as for static light scattering, this technique allows to overcome the limited resolution of DLS and SLS (i.e., of 200 nm or less).

The SALS technique is suitable to study the evolution of microstructure and spatial correlations in complex fluids and soft condensed matter. More specifically, the SALS technique is routinely utilized to investigate the microstructure and spatial correlations in semi-crystalline polymers, and thermotropic liquid crystalline polymers, such as the in situ studies of flowing complex fluids [156,157], polymeric blends, phase separation dynamics and kinetics in fluid mixtures [158,159]. There are also a number of biomedical applications of SALS, including the study of the gross fiber structure of planar connective tissues [160], strain-directed collagen degradation in native tissue and biological cells [161] and the study of termoresponsive biopolymer hydrogels [162].

### 4.2. Dynamic Light Scattering (DLS)

Dynamic light scattering (DLS), also known as quasi-elastic light scattering (QELS) or photon correlation spectroscopy (PCS), furnishes structural and dynamical information of particles in solution, such as biomolecules, nano-colloids, emulsions and gels. In a DLS experiment, the sample under investigation is illuminated by a monochromatic laser beam, while the fluctuations of the scattered light is detected (at a fixed scattering angle θ) by a fast photon detector, and processed by a digital autocorrelator (Figure 12). The Brownian motion of the nano-particles in solution causes a superposition of the light scattered by the different moving particles, that give a Doppler broadening of the incident light, as the macromolecules are in continuous (and random) motion in solution. Analysis of the corresponding fluctuations in the scattered intensity *I*(*k*,*t*) (collected at the detector at a given time *t* and at a fixed wavevector *k* = [(4π*n*)/λ]·sin(θ/2) is performed through a digital autocorrelator, which calculates the scattered intensity *I*(*k*,*t*) (time) correlation function (or second-order correlation function) *g*_2_(*τ*) = <*I*(*t*)·*I*(*t* + *τ*)>/<*I*(*t*)^2^> (expressed as an integral over the product of intensities at time t and at the delayed time (*t* + *τ*)). Assuming the approximation that the scattering is homodyne (i.e., photodetector detects only scattered light) and that the photon counting follows a (statistical) Gaussian behaviour, the g_2_(*k*, *τ*) can be expressed by the Siegert relation [151]:

(16) $$g_{2}\left(k,\;\tau \right)=\frac{\langle I\left(t\right)I\left(t+\tau \right)\rangle }{\langle I{\left(t\right)\rangle }^{2}}=B+\beta \lceil g_{1}{\left(k,\tau \right)\rceil }^{2}$$

where, *g*_1_(*k*,*τ*) = <E*(*k*,*t*)*E*(*k*,*t + τ*)>/<E*(*k*,*t*)*E*(*k*,*t*)> is the normalized autocorrelation function of the scattered electric field E(k,t) (or first-order correlation function) [151,152], B is the baseline (∼1) and β is the coherence factor (that depends on the scattering properties of macromolecules, the detector area and the optical alignment). For monodisperse particles in dilute solution, the electric field correlation function follow an exponential behavior: *g*_1_(*k*,*τ*) = *exp*(−*k*^2^*Dτ*), (where *Γ* = −*Dk*^2^ is decay constant) and yields information on the undergoing macromolecules Brownian motion, through the Stokes–Einstein relation *D* = *k_B_T*/*6πηR_H_*, that expresses the (translational) diffusion coefficient D as a function of the particles hydrodynamic radius *R_H_*, the absolute temperature T, and the solvent viscosity *η* (where k_B_ is the Boltzmann constant). The hydrodynamic radius (*R_H_*), represents then the radius of a hypothetical sphere that would diffuse at the same rate as macromolecules under investigation do. It is worth noticing that the translational diffusion coefficient D is a concentration-dependent quantity, and for this, it should be measured at various concentrations and extrapolated to infinite dilution.

As the theory of DLS is only valid for single scattered light, possible non-negligible contributions from multiple scattering (especially for larger particles at high concentration or with high scattering contrast) can result in large analysis errors. However, it is possible to suppress multiple scattering in DLS by isolating singly scattered light and suppressing undesired contributions from multiple scatterings, through the so called the cross-correlation approach [152,154].

**Figure 14:**
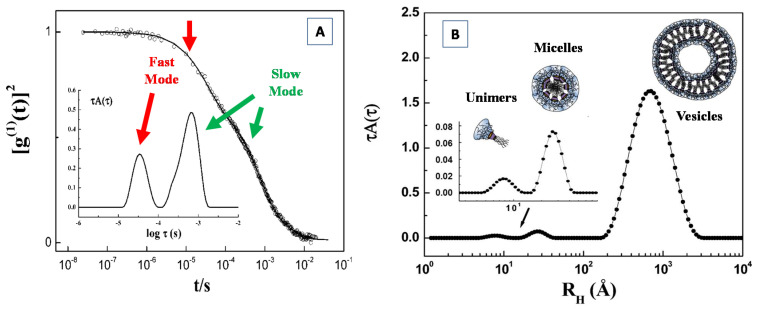
Example of the fit analysis of field autocorrelation function *g_1_*(*k*,*τ*) measured for a system composed of a water solution of amphiphilic modified cyclodextrin (CD). Double exponential fit of the *g*_1_(*k*,*τ*) function for the system CD4 [163] (**A**), and analysis of the relaxation time distribution *τA*(*τ*) as a function of the hydrodynamic radius *R_H_* obtained by the inverse Laplace transformation with regularized positive exponential sum REPES approach (inset of **A**). Triple exponential fit of the *g*_1_(*k*,*τ*) function for the system CD3 (**A**) [163], and the REPES analysis, which evidence the presence of three populations of scattering particles (namely unimers, micelles and vesicels) (**B**).

In Figure 14A we report the scattered field correlation function for a system composed of a water solution of amphiphilic modified cyclodextrin (CD 4) [163], at the temperature *T* = 25 °C and concentration *c* = 1% *w*/*w*. The presence of two relaxation rates (i.e., the fast and slow modes evidenced by the arrows in Figure 14A), are indicative of the presence of two populations of particles with different dimensions. Useful information for the fast and slow contribution can be obtained by means of a double exponential fit of the experimental correlation function, according to following relation

(17) $$g^{\left(1\right)}\left(t\right)=A_{F}exp\left(-k^{2}D_{F}t\right)+A_{S}exp\left(-k^{2}D_{S}t\right)$$

The corresponding diffusion coefficients *D_F_* and *D_S_* have been used to calculate the average hydrodynamic radius from the Stokes−Einstein relation and furnished a value of *R_HF_* = 44 Å, (fast mode), which is connected with the micelles, and a value of *R_HS_* = 570 Å (slow mode) corresponds to the large aggregates.

For a polydisperse system, *g*_1_(*k*, *τ*) cannot be represented as a single exponential decay, but can be expressed as an intensity-weighed integral over a distribution of decay rates *G*(*Γ*) as:

(18) $$g_{1}\left(\tau \right)=\overset{\infty }{\underset{0}{\int }}G(\Gamma )e^{-\Gamma_{\tau }}\;d\Gamma$$

In order to obtain the description of the particles size distribution, the intensity correlation function *g*_1_(*τ*) may be expressed as the Laplace transform of a continuous distribution *G*(*Γ*) of decay times (relaxation rates *Γ*). A variety of different approaches and methods have been developed, in order to perform the inversion of the autocorrelation function and to obtain the distribution of decay rates [164], including regularized positive exponential sum (REPES) [165], constrained regularization program for time inverting noisy signal (CONTIN) [166] and multiexponential non-negative least-squares (NNLS) [167].

In the inset of Figure 14A, the analysis of the relaxation time distribution *τA*(*τ*) as a function of the hydrodynamic radius *R_H_* of the aggregates, as obtained by the inverse Laplace transformation with the REPES approach [164], evidences the distribution of the fast mode and slow mode relaxation corresponding to the field correlation function of Figure 14A. In Figure 14B, we present the scattered field correlation function *g*_1_(*k*,*τ*) for a system composed of a different amphiphilic modified cyclodextrin (CD 3) [163], where the presence of three relaxation rates were put in correspondence with three different populations of scattering particles. Interestingly, a closer inspection of Figure 14B (inset) evidences that, together with vesicles-like aggregates (with *R_H_*_3_ = 650 Å) and small micelles (with *R_H_*_2_ = 28 Å), the investigated system contains the presence of even smaller particles with an average hydrodynamic radius of *R_H_*_1_ ≈ 7.5 Å, which can be attributed to the presence of free molecules in solution (modified cyclodextrin unimer).

A standard technique used to analyze dynamic light-scattering data measured for polydisperse samples is given by the method of cumulants [151,152,153]. According to this approach the autocorrelation function *g*_1_(*k*,*τ*), can be described in terms of a distribution of decay rates. The *g*_1_(*k*,*τ*) is then expanded as the sum of several exponential decay functions (with different decays rates). The cumulants expansion provides, then, information about the average quantities (moments) of this distribution.

(19) $$ln\left|g_{1}\left(k,\tau \right)\right|=-\Gamma_{eff}t+\frac{1}{2}\mu_{2}t^{2}-\frac{1}{3}!\mu_{3}t^{3}+\ldots$$

where *Γ_eff_* is the mean decay rate (which is related to the z-average diffusion coefficient Dz) and *μ_n_* represent the moments of the distribution of the decay rates. The quantity *σ = μ*_2_*/Γ*^2^*_eff_* represents the variance of the distribution (connected with the size polydispersity of the nanoparticles) [151,153].

In conclusion, the derivation of detailed structural and dynamical information by light scattering requires the development of highly performant instruments in combination with even more sophisticated modeling approaches. In this respect, light scattering techniques, in combination with enhanced structural modeling, are expected to have a great impact on future research challenges in biomaterials science.

## 5. Characterization with Complementary Techniques

In order to correlate the self-assembly properties (regulating the composition-structure relationship) obtained by scattering experiments to the underlying biological functions of bio-nanostructured materials, the suitable combination with complementary experimental methods are often necessary. For instance, X-ray crystallography and NMR are powerful structural techniques, when used in combination with SAXS analysis, as the synergistic relationship between those methods provides a better understanding of the bio-nanostructured systems as a whole [168,169]. More specifically, crystallographic and NMR structures analysis may be used to validate the accuracy, on a statistical level, of the molecular envelopes reconstructed from the SAXS data and to compare and highlight complementary structural information provided by SAXS experiments [168,169]. Moreover, while scattering techniques make it possible to characterize the structure and the interactions of biomaterials and biomolecules, from both a quantitative and qualitative point of view, many other complementary techniques can be employed to determine the biomolecular interactions that take place in relevant functional processes, including several spectroscopy techniques such as FT-infrared (FT-IR), ultraviolet (UV), electron spin resonance (ESR), electron paramagnetic resonance (EPR) and circular dichroism, [170,171,172,173]. Furthermore, the combination of complementary highly sensitive techniques of biophysical interest, such as the differential scanning calorimetry (DSC) [174] and isothermal titration calorimetry (ITC) [175], allows for the investigation of the thermodynamic aspects of the morphological (and phase) transition that take place in complex biomaterials within their biological micro-environment.

Particularly interesting is the use of scattering methods in combination with microscopy techniques, such as transmission electron microscopy (TEM), scanning electron microscopy (SEM), atomic force microscopy (AFM), as they provide high-resolution images for studying self-assembly processes, phase transition mechanisms and dynamic phenomena in complex biomaterials systems [176,177,178,179]. Moreover, the structural information in real (direct) space obtained by microscopy techniques can support the structural description of the nanostructured (bio-)materials systems obtained by scattering methods in the reciprocal (Fourier) space. Furthermore, the two techniques can be combined in an efficient way in order to deepen the structural details obtained by the individual techniques. For instance, the electron microscopes frequently have the option of performing an electron diffraction measurement; conversely, the scattering experiments can be performed in real-space mapping modes using micro- or nano-beams [176,177].

While microscopy images are a representation of the direct (real-)space, the scattering data represent a Fourier transform of the real-space distribution, thus, yielding reciprocal-space data that must be analyzed by adopting a structural model of the representative material system. Moreover, microscopy techniques focus rather on a “local” image of the sample, and it is not always straightforward to judge whether the observed microscopy images are truly representative of the entire material system under investigation or if it is an outlier. While, on the other hand, the scattering pattern represents an average description of the structural features of the material probed over the entire illuminated volume, and then it identifies the statistically relevant and dominant structural organization in a sample. Furthermore, many microscopy techniques (such as SEM, AFM, STM) are limited to the surface (2D) characteristics detection or require very thin samples (TEM), except for a few techniques (such in the case of 3D reconstruction via tomography and confocal imaging). On the contrary, the scattering techniques are able to penetrate deeply through the sample to study (from microns to mm depending on materials and radiation probe). This circumstance is a very important factor in the case of biomaterials investigations, as they can facilitate the in situ and in operando experiments [176,177,178]. For instance, biological samples such as lipid vesicles or proteins can be investigated directly in aqueous environments at physiological (or room) temperatures on unmodified samples, whereas the use of electron microscopy typically requires cryo-cooling and/or sectioning of the biological systems. Moreover, special sample preparations are frequently required for microscopy techniques; e.g., generating a thin cross-section material (sample) or casting on particular substrates/holder. Microscopy also frequently requires some manipulations that may perturb the sample, such as staining the sample to improve contrast.

Finally, scattering methods can probe complex highly-ordered structures (otherwise difficult to understand when viewed in projection in microscopy measurements), furnishing a well-defined reciprocal-lattice description, whose symmetry and spacing are clearly identifiable. Moreover, scattering techniques can easily identify (and quantitatively describe) disordered materials (e.g., diffuse scattering), which are difficult to interpret and quantify by using microscopy techniques.

## 6. Concluding Remarks and Future Developments

As future research in material science and biotechnology is moving towards the investigation of increasingly complex phenomena in multicomponent systems, the highly performant and efficient characterization methods, in combination with enhanced protocols for structural modeling, will have a very large impact on all these future scientific efforts. Scattering techniques discussed in this review make it possible to elucidate the structure and dynamics of nanomaterials from sub-nm to micron size scales, and down to sub-millisecond time ranges. With the advent of the high brilliance synchrotron radiation sources and neutron beams with improved power available at research center facilities, a decisive improvement in the simultaneous accessibility to the proper time and length scales is achieved, together with the possibility to follow fast structural transition processes in many different material systems. A major advantage of scattering methods is that they provide the ensemble averaged information under in situ and operando conditions. As a result, they are complementary to various imaging techniques, which reveal more local information. Together with traditional structural characterization, scattering methods provide the basis for examining nanoparticle interactions in solution, and for studying the growth process and conformational changes both in equilibrium situations and transient phenomena. Although many qualitative features can be directly extracted from scattering data, the derivation of detailed structural and dynamical information requires even more sophisticated quantitative modeling. In this respect, scattering techniques, in combination with enhanced protocols for structural modeling and computer simulations, are expected to have a very large impact on all these future scientific challenges. Ultimately, the newly gained insight into the study of complex (bio-) material systems makes it possible to ameliorate the control of structure and functions at the molecular length scales, thus, opening the potential for the development of a large variety of nano-scale biomaterials for advanced technological applications.

## Figures and Tables

**Table 1:** Examples of the SANS and SAXS atomic coherent scattering lengths b_i_ (10^−12^ cm) of relevant elements of the biological systems.

Element	*Z*	*SANS*	*SAXS*
Hydrogen (^1^H)	1	−0.374	0.28
Deuterium (^2^D)	1	0.67	0.28
Carbon (^12^C)	6	0.66	1.69
Nitrogen (^14^N)	7	0.94	1.97
Oxygen (^16^O)	8	0.58	2.25
Aluminium (^27^N)	13	0.35	3.65
Phosphorous (^31^P)	15	0.51	4.23
Sulfur (^32^N)	16	0.28	4.50

**Table 2:** SANS and SAXS Scattering length density [SLD] (10^10^ cm^−2^) for H_2_O and D_2_O molecules.

Molecule	SANS	SAXS
H_2_O	−0.56	9.39
D_2_O	6.73	9.38

## References

[1] Gale P., Steed J. (2012). Supramolecular Chemistry: From Molecules to Nanomaterials.

[2] Grzelczak M., Liz-Marzán L.M., Klajn R. (2019). Stimuli-responsive self-assembly of nanoparticles. Chem. Soc. Rev..

[3] Chircov C., Spoiala A., Păun C., Crăciun L., Ficai D., Ficai A., Andronescu E., Turculeƫ Ș.C. (2020). Mesoporous Silica Platforms with Potential Applications in Release and Adsorption of Active Agents. Molecules.

[4] Liu R., Hudalla G.A. (2019). Using Self-Assembling Peptides to Integrate Biomolecules into Functional Supramolecular Biomaterials. Molecules.

[5] Lombardo D., Calandra P., Magazù S., Wanderlingh U., Barreca D., Pasqua L., Kiselev M.A. (2018). Soft nanoparticles charge expression within lipid membranes: The case of amino terminated dendrimers in bilayers vesicles. Colloids Surf. B Biointerfaces.

[6] Tiwari A., Tiwari A. (2013). Nanomaterials in Drug Delivery, Imaging, and Tissue Engineerin.

[7] Lombardo D., Kiselev M.A., Magazu S., Calandra P. (2015). Amphiphiles Self-Assembly: Basic Concepts and Future Perspectives of Supramolecular Approaches. Adv. Condens. Matter Phys..

[8] Ilie A., Ghiţulică C., Andronescu E., Cucuruz A., Ficai A. (2016). New composite materials based on alginate and hydroxyapatite as potential carriers for ascorbic acid. Int. J. Pharm..

[9] Wang L., Gong C., Yuan X., Wei G. (2019). Controlling the Self-Assembly of Biomolecules into Functional Nanomaterials through Internal Interactions and External Stimulations: A Review. Nanomaterials.

[10] Salehi B., Calina D., Docea A.O., Koirala N., Aryal S., Lombardo D., Pasqua L., Taheri Y., Castillo C.M.S., Martorell M. (2020). Curcumin’s Nanomedicine Formulations for Therapeutic Application in Neurological Diseases. J. Clin. Med..

[11] Kaga S., Truong N.P., Esser L., Senyschyn D., Sanyal A., Sanyal R., Quinn J.F., Davis T.P., Kaminskas L.M., Whittaker M.R. (2017). Influence of Size and Shape on the Biodistribution of Nanoparticles Prepared by Polymerization-Induced Self-Assembly. Biomacromolecules.

[12] Lanza R., Langer R., Vacanti J., Atala A. (2020). Principles of Tissue Engineering.

[13] Mantha S., Pillai S., Khayambashi P., Upadhyay A., Zhang Y., Tao O., Pham H.M., Tran S.D. (2019). Smart Hydrogels in Tissue Engineering and Regenerative Medicine. Materials.

[14] Radulescu M., Ficai D., Oprea O., Ficai A., Andronescu E., Holban A.M. (2015). Antimicrobial Chitosan based formulations with impact on different biomedical applications. Curr. Pharm. Biotechnol..

[15] Chimisso V., Aleman Garcia M.A., Yorulmaz Avsar S., Dinu I.A., Palivan C.G. (2020). Design of Bio-Conjugated Hydrogels for Regenerative Medicine Applications: From Polymer Scaffold to Biomolecule Choice. Molecules.

[16] Aguilar Z.P. (2013). Nanomaterials for Medical Applications.

[17] Doshi N., Mitragotri S. (2009). Designer Biomaterials for Nanomedicine. Adv. Funct. Mater..

[18] Andronescu E., Ficai M., Voicu G., Ficai D., Maganu M., Ficai A. (2010). Synthesis and characterization of collagen/hydroxyapatite: Magnetite composite material for bone cancer treatment. J. Mater. Sci. Mater. Med..

[19] Pasqua L., De Napoli I.E., De Santo M., Greco M., Catizzone E., Lombardo D., Montera G., Comandè A., Nigro A., Morelli C. (2019). Mesoporous silica-based hybrid materials for bone-specific drug delivery. Nanoscale Adv..

[20] Gong C., Sun S., Zhang Y., Sun L., Su Z., Wu A., Wei G. (2019). Hierarchical nanomaterials via biomolecular self-assembly and bioinspiration for energy and environmental applications. Nanoscale.

[21] Malmsten M. (2006). Surfactants and Polymers in Drug Delivery.

[22] Lombardo D., Calandra P., Pasqua L., Magazù S. (2020). Self-assembly of Organic Nanomaterials and Biomaterials: The Bottom-Up Approach for Functional Nanostructures Formation and Advanced Applications. Materials.

[23] Jaekel A., Stegemann P., Saccà B. (2019). Manipulating Enzymes Properties with DNA Nanostructures. Molecules.

[24] Russina O., Gontrani L., Fazio B., Lombardo D., Triolo A., Caminiti R. (2010). Selected chemical–physical properties and structural heterogeneities in 1-ethyl-3-methylimidazolium alkyl-sulfate room temperature ionic liquids. Chem. Phys. Lett..

[25] Lehn J.-M. (1995). Supramolecular Chemistry.

[26] Lee Y.S. (2008). Self-Assembly and Nanotechnology, a Force Balance Approach.

[27] Calandra P., Caschera D., Liveri V.T., Lombardo D. (2015). How self-assembly of amphiphilic molecules can generate complexity in the nanoscale. Colloids Surf. A Physicochem. Eng. Asp..

[28] Ariga K., Ito M., Mori T., Watanabe S., Takeya J. (2019). Atom/molecular nanoarchitectonics for devices and related applications. Nano Today.

[29] Pica A., Guran C., Andronescu E., Oprea O., Ficai D., Ficai A. (2012). Antimicrobial performances of some film forming materials based on silver nanoparticles. J. Optoelectron. Adv. Mater..

[30] Salam A., Makhlouf H., Barhoum A. (2018). DNA Nanostructures: Chemistry, Self-Assembly, and Applications. Emerging Applications of Nanoparticles and Architecture Nanostructures.

[31] Feigin L.A., Svergun D.I. (1987). Structure Analysis by Small-Angle X-ray and Neutron Scattering.

[32] Glatter O., Kratky O. (1982). Small-Angle X-ray Scattering.

[33] Squires G.L. (1997). Introduction to the Theory of Thermal Neutron Scattering.

[34] Fitter J., Gutberlet T., Katsaras J. (2006). Neutron Scattering in Biology Techniques and Applications.

[35] (2009). X-ray Data Booklet, A.C..

[36] Stuhrmanna H.B. (2007). Contrast variation in X-ray and neutron scattering. J. Appl. Cryst..

[37] Luo G., Zhang Q., Del Castillo A.R., Urban V., O’Neill H. (2009). Characterization of sol gel-encapsulated proteins using small-angle neutron scattering. ACS Appl. Mater. Interfaces.

[38] Jacrot B. (1976). Study of biological structures by neutron-scattering from solution. Rep. Prog. Phys..

[39] Sztucki M., Di Cola E., Narayanan T. (2012). Anomalous small-angle X-ray scattering from charged soft matter. Eur. Phys. J. Spec. Top..

[40] Gebel G. (2013). Structure of Membranes for Fuel Cells: SANS and SAXS Analyses of Sulfonated PEEK Membranes and Solutions. Macromolecules.

[41] Varga Z., Bóta A., Goerigk G. (2006). Localization of Dibromophenol in DPPC/Water Liposomes Studied by Anomalous Small-Angle X-ray Scattering. J. Phys. Chem. B.

[42] Ballauff M., Jusufi A. (2006). Anomalous small-angle X-ray scattering: Analyzing correlations and fluctuations in polyelectrolytes. Colloid Polym. Sci..

[43] Nakasako M., Wada M., Tokutomi S., Yamamoto K., Sakai J., Kataoka M., Tokunaga F., Furuya M. (1990). Quaternary structure of a PEA phytochrome I dimer studied with small-angle X-ray scattering and rotatory shadowing electron microscopy. Photochem. Photobiol..

[44] Dingenouts N., Ballauff M. (1993). Small-angle x-ray analysis of latex particles using contrast variation. Acta Polym..

[45] Kiselev M.A., Lesieur P., Kisselev A.M., Lombardo D., Killany M., Lesieur S., Ollivon M. (2001). A sucrose solutions application to the study of model biological membranes. Nucl. Instrum. Methods Phys. Res. A.

[46] Kiselev M., Lesieur P., Kisselev A.M., Lombardo D., Killany M., Lesieur S. (2001). Sucrose solutions as prospective medium to study the vesicle structure: SAXS and SANS study. J. Alloys Compd..

[47] Naruse K., Eguchi K., Akiba I., Sakurai K. (2009). Hiroyasu, Flexibility and Cross-Sectional Structure of an Anionic Dual-Surfactant Wormlike Micelle Explored with Small-Angle X-ray Scattering Coupled with Contrast Variation Technique. J. Phys. Chem. B.

[48] Hickl P., Ballauff M., Jada A. (1996). Small-Angle X-ray Contrast-Variation Study of Micelles Formed by Poly (styrene)−Poly (ethylene oxide) Block Copolymers in Aqueous Solution. Macromolecules.

[49] Bolze J., Hörner K.D., Ballauff M. (1996). Adsorption of the Nonionic Surfactant Triton X-405 on Polystyrene Latex Particles As Monitored by Small-Angle X-ray Scattering. Langmuir.

[50] Fernandez R.M., Riske K.A., Amaral L.Q., Itri R., Lamy M.T. (2008). Influence of salt on the structure of DMPG studied by SAXS and optical microscopy. Biochim. Biophys. Acta.

[51] Schmidt P.W., Brumberger H. (1995). Modern Aspects of Small-Angle Scattering.

[52] Di Cola E., Grillo I., Ristori S. (2016). Small Angle X-ray and Neutron Scattering: Powerful Tools for Studying the Structure of Drug-Loaded Liposomes. Pharmaceutics.

[53] Zemb T., Lindner P. (2002). Neutron, X-rays and Light Scattering Methods Applied to Soft Condensed Matter.

[54] Kiselev M.A., Lombardo D. (2017). Structural characterization in mixed lipid membrane systems by neutron and X-ray scattering. Biochim. Biophys. Acta Gen. Subj..

[55] Alexandridis P., Lindman B. (2000). Amphiphilic Block Copolymers: Self-Assembly and Applications (Studies in Surface Science and Catalysis).

[56] Mallamace F., Beneduci R., Gambadauro P., Lombardo D., Chen S.H. (2001). Glass and percolation transitions in dense attractive micellar system. Phys. A Stat. Mech. Appl..

[57] Chen S.H., Mallamace F., Faraone A., Gambadauro P., Lombardo D., Chen W.R. (2002). Observation of a re-entrant kinetic glass transition in a micellar system with temperature-dependent attractive interaction. Eur. Phys. J. E Soft Matter..

[58] Mai Y., Eisenberg A. (2012). Self-Assembly of Block Copolymers. Chem. Soc. Rev..

[59] Nelemans L.C., Gurevich L. (2020). Drug Delivery with Polymeric Nanocarriers-Cellular Uptake Mechanisms. Materials.

[60] Hamley I., Castelletto V., Borsali R., Pecora R. (2008). Small-Angle Scattering of Block Copolymers.

[61] Lombardo D., Munaò M., Calandra P., Pasqua L., Caccamo M.T. (2019). Evidence of pre-micellar aggregates in water solution of amphiphilic PDMS-PEO block copolymer. Phys. Chem. Chem. Phys..

[62] Narayanan T., Sztucki M., Van Vaerenbergh P., Léonardon J., Gorini J., Claustre L., Sever F., Morse J., Boesecke P. (2018). A multipurpose instrument for time-resolved ultra-small-angle and coherent X-ray scattering. J. Appl. Crystallogr..

[63] Amenitsch H., Bernstorff S., Kriechbaum M., Lombardo D., Mio H., Rappolt M., Laggner P. (1997). Performance and first results of the ELETTRA high-flux beamline for small-angle X-ray scattering. J. Appl. Cryst..

[64] Lobry L., Micali N., Mallamace F., Liao C., Chen S.H. (1999). Interaction and percolation in the L64 triblock copolymer micellar system. Phys. Rev. E.

[65] Pedersen J.S., Gerstenberg M.C. (1996). Scattering Form Factor of Block Copolymer Micelles. Macromolecules.

[66] Kotlarchyk M., Chen S.-H. (1983). Analysis of small angle neutron scattering spectra from polydisperse interacting colloids. J. Chem. Phys..

[67] Kiselev M.A., Janich M., Hildebrand A., Strunz P., Neubert R.H.H., Lombardo D. (2013). Structural transition in aqueous lipid/bile salt [DPPC/NaDC] supramolecular aggregates: SANS and DLS study. Chem. Phys..

[68] Svergun D.I., Semenyuk A.V., Feigin L.A. (1988). Small-angle-scattering-data treatment by the regularization method. Acta Cryst..

[69] Svergun D.I., Koch M.H.J. (2003). Small-angle scattering studies of biological macromolecules in solution. Rep. Prog. Phys..

[70] Weyerich B., Brunner-Popela J., Glatter O. (1999). Small-angle scattering of interacting particles. II. Generalized indirect Fourier transformation under consideration of the effective structure factor for polydisperse systems. J. Appl. Crystallogr..

[71] Szymusiak M., Kalkowski J., Luo H. (2017). Core-shell Structure and Aggregation Number of Micelles Composed of Amphiphilic Block Copolymers and Amphiphilic Heterografted Polymer Brushes Determined by Small-Angle X-ray Scattering. ACS Macro Lett..

[72] Glatter O. (1979). The Interpretation of Real-Space Information from Small-Angle Scattering Experiments. J. Appl. Crystallogr..

[73] Heftberger P., Kollmitzer B., Heberle F.A., Pan J., Rappolt M., Amenitsch H., Kučerka N., Katsaras J., Pabst G. (2013). Global small-angle X-ray scattering data analysis for multilamellar vesicles: The evolution of the scattering density profile model. J. Appl. Crystallogr..

[74] Koch M.H., Vachette P., Svergun D.I. (2003). Small-angle scattering: A view on the properties, structures and structural changes of biological macromolecules in solution. Q. Rev. Biophys..

[75] Calandra P., Ruggirello A., Mele A., Liveri V.T. (2010). Self-assembly in surfactant-based liquid mixtures: Bis(2-ethylhexyl)phosphoric acid/bis(2-ethylhexyl)amine systems. J. Colloid Interface Sci..

[76] Micali N., Scolaro L.M., Romeo A., Lombardo D., Lesieur P., Mallamace F. (1998). Structural properties of methanol-polyamidoamine dendrimer solutions. Phys. Rev. E.

[77] Lesieur P., Kiselev M.A., Barsukov L.I., Lombardo D. (2000). Temperature-induced micelle to vesicle transition: Kinetic effects in the DMPC/NaC system. J. Appl. Cryst..

[78] Winter R., Koehling R. (2004). Static and time-resolved synchrotron small-angle x-ray scattering studies of lyotropic lipid mesophases, model biomembranes and proteins in solution. J. Condens. Matter Phys..

[79] Chaudhuri B., Muñoz I.G., Qian S., Urban V.S. (2017). Biological Small Angle Scattering: Techniques, Strategies and Tips.

[80] Calandra P., Liveri V.T., Riello P., Freris I., Mandanici A. (2012). Self-assembly in surfactant-based liquid mixtures: Octanoic acid/Bis(2-ethylhexyl)amine systems. J. Colloid Interface Sci..

[81] Lombardo D. (2009). Liquid-like ordering of negatively charged poly (amidoamine) (PAMAM) dendrimers in solution. Langmuir.

[82] Kiselev M., Lesieur P., Kisselev A.M., Lombardo D., Aksenov V. (2002). Model of separated form factors for unilamellar vesicles. Appl. Phys. A.

[83] Holmberg K., Jonsson B., Kronberg B., Lindman B. (2002). Surfactants and Polymers in Aqueous Solution.

[84] Laughlin R.G. (1994). The Aqueous Phase Behavior of Surfactants.

[85] Yamada M., Honma I.J. (2004). Anhydrous protonic conductivity of a self-assembled acid−base composite material. J. Phys. Chem. B.

[86] Kim J.D., Honma I. (2005). Anhydrous solid state proton conductor based on enzimidazole/monododecyl phosphate molecular hybrids. Solid State Ion..

[87] Calandra P., Turco Liveri V., Ruggirello A.M., Licciardi M., Lombardo D., Mandanici A. (2015). Anti-Arrhenian behaviour of conductivity in octanoic acid–bis (2-ethylhexyl) amine systems: A physico-chemical study. J. Mater. Chem. C.

[88] Pochylski M., Lombardo D., Calandra P. (2019). Optical Birefringence Growth Driven by Magnetic Field in Liquids: The Case of Dibutyl Phosphate/Propylamine System. Appl. Sci..

[89] Rikken R., Kerkenaar H., Nolte R., Maan J., Van Hest J., Christianen P., Wilson D. (2014). Probing morphological changes in polymersomes with magnetic birefringence. Chem. Commun..

[90] Calandra P., Caputo P., De Santo M.P., Todaro L., Turco Liveri V., Rossi C.O. (2019). Effect of additives on the structural organization of asphaltene aggregates in bitumen. Constr. Build. Mater..

[91] Turco Liveri V., Lombardo D., Pochylski M., Calandra P. (2018). Molecular association of small amphiphiles: Origin of ionic liquid properties in dibutyl phosphate/propylamine binary mixtures. J. Mol. Liq..

[92] Calandra P. (2020). On the physico-chemical basis of self-nanosegregation giving magnetically-induced birefringence in dibutyl phosphate/bis(2-ethylhexyl) amine systems. J. Mol. Liq..

[93] Cho H.S., Dashdorj N., Schotte F., Graber T., Henning R., Anfinrud P. (2010). Protein structural dynamics in solution unveiled via 100-ps time-resolved x-ray scattering. Proc. Natl. Acad. Sci. USA.

[94] Pollack L. (2011). Time resolved SAXS and RNA folding. Biopolymers.

[95] Bruetzel L.K., Walker P.U., Gerling T., Dietz H., Lipfert J. (2018). Time-Resolved Small-Angle X-ray Scattering Reveals Millisecond Transitions of a DNA Origami Switch. Nano Lett..

[96] Narayanan T., Wacklin H., Konovalov O., Lund R. (2017). Recent applications of synchrotron radiation and neutrons in the study of soft matter. Crystallogr. Rev..

[97] Amenitsch H., Rappolt M., Kriechbaum M., Mio H., Laggner P., Bernstorff S. (1998). First performance assessment of the small-angle X-ray scattering beamline at ELETTRA. J. Synchrotron Radiat..

[98] Möller J., Léonardon J., Gorini J., Dattani R., Narayanan T. (2016). A sub-ms pressure jump setup for time-resolved X-ray scattering. Rev. Sci. Instrum..

[99] Kiselev M.A. (2011). Methods for lipid nanostructure investigation at neutron and synchrotron sources. Phys. Part. Nucl..

[100] Ludescher L., Morak R., Balzer C., Waag A.M., Braxmeier S., Putz F., Busch S., Gor G.Y., Neimark A.V., Husing N. (2019). In Situ Small-Angle Neutron Scattering Investigation of Adsorption-Induced Deformation in Silica with Hierarchical Porosity. Langmuir.

[101] Bonaccorsi L., Lombardo D., Longo A., Proverbio E., Triolo A. (2009). Dendrimer template directed self-assembly during zeolite formation. Macromolecules.

[102] Gommes C.J., Prieto G., de Jongh P.E. (2016). Small-Angle Scattering Analysis of Empty or Loaded Hierarchical Porous MaterialsJ. Phys. Chem. C.

[103] Bonaccorsi L., Calandra P., Amenitsch H., Proverbio E., Lombardo D. (2013). Growth of fractal aggregates during template directed SAPO-34 zeolite formation. Micropor. Mesopor. Mater..

[104] Huang X., Zheng S., Kim I. (2014). Hyperbranched Polymers and Dendrimers as Templates for Organic/Inorganic Hybrid Nanomaterials. J. Nanosci. Nanotechnol..

[105] Sanchez C., Belleville P., Popall M., Nicole L. (2011). Applications of advanced hybrid organic-inorganic nanomaterials: From laboratory to market. Chem. Soc. Rev..

[106] Michaux F., Baccile N., Imperor-Clerc M., Malfatti L., Folliet N., Gervais C., Manet S., Meneau F., Pedersen J.S., Babonneau F. (2012). In situ time-resolved SAXS study of the formation of mesostructured organically modified silica through modeling of micelles evolution during surfactant-templated self-assembly. Langmuir.

[107] Bonaccorsi L., Calandra P., Kiselev M.A., Amenitsch H., Proverbio E., Lombardo D. (2013). Self-assembly in poly(dimethylsiloxane)-poly(ethylene oxide) block copolymer template directed synthesis of linde type A zeolite. Langmuir.

[108] Teixeira J. (1988). Small-Angle Scattering by Fractal Systems. J. Appl. Cryst..

[109] Hunter R.J. (1986). Foundations of Colloid Science.

[110] Hansen J.P., Mc Donald I.A. (1986). Theory of Simple Liquids.

[111] Caccamo C. (1996). Integral Equation Theory Description of Phase Equilibria in Classical Fluids. Phys. Rep..

[112] Lombardo D. (2014). Modeling Dendrimers Charge Interaction in Solution: Relevance in Biosystems. Biochem. Res. Int..

[113] Sato K., Anzai J. (2013). Dendrimers in layer-by-layer assemblies: Synthesis and applications. Molecules.

[114] Mhlwatika Z., Aderibigbe B.A. (2018). Application of Dendrimers for the Treatment of Infectious Diseases. Molecules.

[115] Lombardo D., Calandra P., Bellocco E., Laganà G., Barreca D., Magazù S., Wanderlingh U., Kiselev M.A. (2016). Effect of anionic and cationic polyamidoamine (PAMAM) dendrimers on a model lipid membrane. Biochim. Biophys. Acta Biomembr..

[116] Ekimoto T., Kokabu Y., Oroguchi T., Ikeguchi M. (2019). Combination of coarse-grained molecular dynamics simulations and small-angle X-ray scattering experiments. Biophys. Physicobiol..

[117] Björling A., Niebling S., Marcellini M., Van Der Spoel D., Westenhoff S. (2015). Deciphering Solution Scattering Data with Experimentally Guided Molecular Dynamics Simulations. J. Chem. Theory Comput..

[118] Sachs J., Petrache H., Woolf T. (2003). Interpretation of small angle X-ray measurements guided by molecular dynamics simulations of lipid bilayers. Chem. Phys. Lipids.

[119] Pan J., Cheng X., Monticelli L., Heberle F.A., Kučerka N., Tieleman D.P., Katsara S.J. (2014). The molecular structure of a phosphatidylserine bilayer determined by scattering and molecular dynamics simulations. Soft Matter.

[120] Arbe A., Alvareza F., Colmenero J. (2012). Neutron scattering and molecular dynamics simulations: Synergetic tools to unravel structure and dynamics in polymers. Soft Matter.

[121] Alford A., Kozlovskaya V., Kharlampieva E. (2017). Small Angle Scattering for Pharmaceutical Applications: From Drugs to Drug Delivery Systems. Adv. Exp. Med. Biol..

[122] Narayanan T. (2008). Synchrotron small-angle X-ray scattering. Soft Matter: Characterization.

[123] Kiselev M., Zemlyanaya E., Ryabova N.Y., Hauss T., Almásy L., Funari S., Zbytovská J., Lombardo D. (2014). Influence of ceramide on the internal structure and hydration of the phospholipid bilayer studied by neutron and X-ray scattering. Appl. Phys. A.

[124] Schilt Y., Berman T., Wei X., Barenholz Y., Raviv U. (2016). Using solution X-ray scattering to determine the high-resolution structure and morphology of PEGylated liposomal doxorubicin nanodrugs. Biochim. Biophys. Acta.

[125] Yaghmur A., Rappolt M., Jonassen A.L., Schmitt M., Larsen S. (2020). In situ monitoring of the formation of lipidic non-lamellar liquid crystalline depot formulations in synovial fluid. J. Colloid Interface Sci..

[126] Giulimondi F., Digiacomo L., Pozzi D., Palchetti S., Vulpis E., Capriotti A.L., Chiozzi R.Z., Laganà A., Amenitsch H., Masuelli L. (2019). Interplay of protein corona and immune cells controls blood residency of liposomes. Nat. Commun..

[127] Kennedy M.T., Pozharski E.V., Rakhmanova V.A., MacDonald R.C. (2000). Factors governing the assembly of cationic phospholipid-DNA complexes. Biophys. J..

[128] Simberg D., Danino D., Talmon Y., Minsky A., Ferrari M.E., Wheeler C.J., Barenholz Y. (2001). Phase behavior, DNA ordering, and size instability of cationic lipoplexes Relevance to optimal transfection activity. J. Biol. Chem..

[129] Lombardo D., Calandra P., Caccamo M.T., Magazù S., Kiselev M.A. (2019). Colloidal stability of liposomes. AIMS Mater. Sci..

[130] Schmidt N.W., Mishra A., Wang J., DeGrado W.F., Wong G.C. (2013). Influenza virus A M2 protein generates negative Gaussian membrane curvature necessary for budding and scission. J. Am. Chem. Soc..

[131] Schmidt N., Wong G. (2013). Antimicrobial peptides and induced membrane curvature: Geometry, coordination chemistry, and molecular engineering. Curr. Opin. Solid State Mater. Sci..

[132] Shai Y. (1999). Mechanism of the binding, insertion and destabilization of phospholipid bilayer membranes by A-helical antimicrobial and cell non-selective membrane-lytic peptides. Biochim. Biophys. Acta Biomembr..

[133] Lombardo D., Caccamo M.T., Magazù S., Kiselev M.A., Calandra P. (2019). Enhancement of colloidal stability of drug nanocarriers in complex biological environment. AAPP Phys. Math. Nat. Sci..

[134] Pozzi D., Colapicchioni V., Caracciolo G., Piovesana S., Capriotti A.L., Palchetti S., De Grossi S., Riccioli A., Amenitsch H., Laganà A. (2014). Effect of polyethyleneglycol (PEG) chain length on the bio–nano-interactions between PEGylated lipid nanoparticles and biological fluids: From nanostructure to uptake in cancer cells. Nanoscale.

[135] Lombardo D., Calandra P., Caccamo M.T., Magazù S., Pasqua L., Kiselev M.A. (2020). Interdisciplinary approaches to the study of biological membranes. AIMS Biophys..

[136] Franke D., Jeffries C.M., Svergun D.I. (2018). Machine Learning Methods for X-Ray Scattering Data Analysis from Biomacromolecular Solutions. Biophys. J..

[137] Frewein M.P., Rumetshofer M., Pabst G. (2019). Global small-angle scattering data analysis of inverted hexagonal phases. J. Appl. Crystallogr..

[138] Putnam C.D., Hammel M., Hura G.L., Tainer J.A. (2007). X-ray solution scattering (SAXS) combined with crystallography and computation: Defining accurate macromolecular structures, conformations and assemblies in solution. Q. Rev. Biophys..

[139] Tria G., Mertens H.D., Kachala M., Svergun D.I. (2015). Advanced ensemble modelling of flexible macromolecules using X-ray solution scattering. IUCrJ.

[140] Bernadó P., Svergun D.I. (2012). Structural analysis of intrinsically disordered proteins by small-angle X-ray scattering. Mol. Biosyst..

[141] Bernadó P., Mylonas E., Petoukhov M.V., Blackledge M., Svergun D. (2007). Structural characterization of flexible proteins using small-angle X-ray scattering. J. Am. Chem. Soc..

[142] Hura G.L., Menon A.L., Hammel M., Rambo R.P., Ii F.L.P., Tsutakawa S.E., Jr F.E.J., Classen S., Frankel K.A., Hopkins R.C. (2009). Robust, high-throughput solution structural analyses by small angle X-ray scattering (SAXS). Nat. Methods.

[143] von Gundlach A.R., Garamus V.M., Gorniak T., Davies H.A., Reischl M., Mikut R., Hilpert K., Rosenhahn A. (2016). Small angle X-ray scattering as a high-throughput method to classify antimicrobial modes of action. Biochim. Biophys. Acta.

[144] Hyland L.L., Taraban M., Yu Y.B. (2013). Using Small-Angle Scattering Techniques to Understand Mechanical Properties of Biopolymer-Based Biomaterials. Soft Matter.

[145] Hule R.A., Nagarkar R.P., Hammouda B., Schneider J.P., Pochan D.J. (2009). Dependence of Self-Assembled Peptide Hydrogel Network Structure on Local Fibril Nanostructure. Macromolecules.

[146] Pedersen J.S., Shurtenberger P. (1996). Scattering Functions of Semiflexible Polymers with and without Excluded Volume Effects. Macromolecules.

[147] MacKintosh F.C., Käs J., Janmey P.A. (1995). Elasticity of semiflexible biopolymer networks. Phys. Rev. Lett..

[148] Taraban M.B., Feng Y., Hammouda B., Hyland L.L., Yu Y.B. (2012). Chirality-Mediated Mechanical and Structural Properties of Oligopeptide Hydrogels. Chem. Mater..

[149] Hyland L.L., Taraban M.B., Hammouda B., Bruce Yu Y. (2011). Mutually reinforced multicomponent polysaccharide networks. Biopolymers.

[150] Hyland L.L., Taraban M.B., Feng Y., Hammouda B., Yu Y.B. (2012). Viscoelastic properties and nanoscale structures of composite oligopeptide-polysaccharide hydrogels. Biopolymers.

[151] Berne B.J., Pecora R. (1976). Dynamic Light Scattering.

[152] Brown W. (1996). Light Scattering: Principles and Developmen.

[153] Chu B. (1991). Light Scattering. Basic Principle and Practice.

[154] Lombardo D., Micali N., Villari V., Kiselev M.A. (2004). Large structures in diblock copolymer micellar solution. Phys. Rev. E.

[155] Alexander M., Ross F. (1999). Hallett Small-angle light scattering: Instrumental design and application to particle sizing. Appl. Opt..

[156] Chen S.H., Lombardo D., Mallamace F., Micali N., Trusso S., Vasi C., Laggner P., Glatter O. (1993). Small-angle light scattering in microemulsions (spinodal decomposition). Trends in Colloid and Interface Science VII.

[157] Castelletto V., Hamley I.W. (2006). Capillary flow behavior of worm-like micelles studied by small-angle X-ray scattering and small angle light scattering. Polym. Adv. Technol..

[158] Butler M.F. (2002). Mechanism and kinetics of phase separation in a gelatin/maltodextrin mixture studied by small-angle light scattering. Biomacromolecules.

[159] Sakurai S., Izumitani T., Hasegawa H., Hashimoto T., Han C.C. (1991). Small-angle neutron scattering and light scattering study on the miscibility of poly(styrene-ran-butadiene)/polybutadiene blends. Macromolecules.

[160] Sacks M.S., Smith D.B., Hiester E.D. (1997). A small angle light scattering device for planar connective tissue microstructural analysis. Ann. Biomed. Eng..

[161] Robitaille M.C., Zareian R., DiMarzio C.A., Wan C.-T., Ruberti J.W. (2011). Small-angle light scattering to detect strain-directed collagen degradation in native tissue. Interface Focus..

[162] Dashtimoghadam E., Mirzadeh H., Taromia F.A., Nyström B. (2014). Thermoresponsive biopolymer hydrogels with tunable gel characteristics. RSC Adv..

[163] Lombardo D., Longo A., Darcy R., Mazzaglia A. (2004). Structural Properties of Nonionic Cyclodextrin Colloids in Water. Langmuir.

[164] Stepanek P., Brown W. (1993). Data analysis in dynamic light scattering. Dynamic Light Scattering: The Method and Some Applications.

[165] Jakes J. (1995). Regularized Positive Exponential Sum (REPES) Program—A Way of Inverting Laplace Transform Data Obtained by Dynamic Light Scattering. Collect. Czechoslov. Chem. Commun..

[166] Provencher S.W. (1982). CONTIN: A general purpose constrained regularization program for inverting noisy linear algebraic and integral equations. Comput Phys Commun.

[167] Ruhe A. (1980). Fitting empirical data by positive sums of exponentials. SIAM J. Sci. Stat. Comput..

[168] Grant T.D., Luft J.R., Wolfley J.R., Tsuruta H., Martel A., Montelione G.T., Snell E.H. (2011). Small angle X-ray scattering as a complementary tool for high-throughput structural studies. Biopolymers.

[169] Narayanan T., Konovalov O. (2020). Synchrotron Scattering Methods for Nanomaterials and Soft Matter Research. Materials.

[170] Nieh M.P., Heberle F.A., Katsaras J. (2019). Characterization of Biological Membranes Structure and Dynamics.

[171] Pignatello R., Musumeci T., Basile L. (2011). Biomembrane models and drug-biomembrane interaction studies: Involvement in drug design and development. J. Pharm. Bioallied. Sci..

[172] Mazzaglia A., Angelini N., Darcy R., Donohue R., Lombardo D., Micali N., Sciortino M.T., Villari V., Scolaro L.M. (2003). Novel heterotopic colloids of anionic porphyrins entangled in cationic amphiphilic cyclodextrins: Spectroscopic investigation and intracellular delivery. Chem. Eur. J..

[173] Cretu C., Maiuolo L., Lombardo D., Szerb E.I., Calandra P. (2020). Luminescent Supramolecular Nano-or Microstructures Formed in Aqueous Media by Amphiphile-Noble Metal Complexes. J. Nanomater..

[174] Pignatello R. (2013). Drug-Biomembrane Interaction Studies, the Application of Calorimetric Techniques.

[175] Abraham T., Lewis R.N.A.H., Hodges R.S., McElhaney R.N. (2005). Isothermal titration calorimetry studies of the binding of a rationally designed analogue of the antimicrobial peptide gramicidin s to phospholipid bilayer membranes. Biochemistry.

[176] Bozzola J.J., Russell L.D. (1998). Electron Microscopy: Principles and Techniques for Biologists.

[177] Hayat M. (2000). Principles and Techniques of Electron Microscopy: Biological Applications.

[178] Helvig S., Azmi I.D.M., Moghimi S.M., Yaghmur A. (2015). Recent advances in cryo-TEM imaging of soft lipid nanoparticles. AIMS Biophys..

[179] Variola F. (2015). Atomic force microscopy in biomaterials surface science. Phys. Chem. Chem. Phys..

